# Pairing of homologous chromosomes in *C. elegans* meiosis requires DEB-1 - an orthologue of mammalian vinculin

**DOI:** 10.1080/19491034.2019.1602337

**Published:** 2019-05-08

**Authors:** Jana Rohožková, Lenka Hůlková, Jana Fukalová, Petr Flachs, Pavel Hozák

**Affiliations:** a Department of Epigenetics of the Cell Nucleus, Institute of Molecular Genetics AS CR, v.v.i. division BIOCEV, Vestec, Czech Republic; b Department of Biology of the Cell Nucleus, Institute of Molecular Genetics AS CR, v.v.i., Prague, Czech Republic; c Microscopy centre, Institute of Molecular Genetics AS CR, v.v.i., Prague, Czech Republic

**Keywords:** DEB-1, LINC complex, chromosome pairing, prophase I, *C. elegans*, vinculin

## Abstract

During meiosis, homologous chromosomes undergo a dramatic movement in order to correctly align. This is a critical meiotic event but the molecular properties of this ‘chromosomal dance’ still remainunclear. We identified DEB-1 – an orthologue of mammalian vinculin – as a new component of the mechanistic modules responsible for attaching the chromosomes to the nuclear envelope as apart of the LINC complex. In early meiotic nuclei of *C. elegans*, DEB-1 is localized to the nuclear periphery and alongside the synaptonemal complex of paired homologues. Upon DEB-1 depletion, chromosomes attached to SUN-1 foci remain highly motile until late pachytene. Although the initiation of homologue pairing started normally, irregularities in the formation of the synaptonemal complex occur, and these results in meiotic defects such as increased number of univalents at diakinesis and high embryonic lethality. Our data identify DEB-1 as a new player regulating chromosome dynamics and pairing during meiotic prophase I.

## Introduction

Meiosis is a complex process in which diploid cells give rise to haploid gametes necessary for sexual reproduction of eukaryotes. In most species, gametogenesis is accompanied by the establishment of homologous pairs, formation of the synaptonemal complex (SC), and cross-over recombination [1]. Also in *Caenorhabditis elegans*, chromosomes undergo all stages of meiotic prophase in preparation for meiosis I and the reducing division. In both males and hermaphrodites, germ-cell nuclei are arranged in zones along the distal-proximal axis of adult gonads. As nuclei enter the prophase preceding the first meiotic division, homologous chromosomes partially condense and pair. Chromosome pairing and modification of nuclear organization begin in the transition zone (corresponds to the leptotene and zygotene stages; further termed also as leptotene/zygotene) accompanied by the reorganization of chromosomes toward one side of the nucleus. In the pachytene stage, the pairing process is completed and the synapsed chromosomes become dispersed about the nuclear periphery. When meiocytes finally exit the pachytene, the SC disassemblies and chromosomes align into a bipolar orientation on the meiotic spindle. As meiotic chromosomes progress from diplotene to diakinesis, they become highly condensed, forming six discrete oocyte bivalents.

During the pairing process in the transition zone of the worm gonad, chromosomes undergo a dynamic movement, involving association of chromosome ends with cytoskeletal motion-generating machineries in early prophase. The cytoskeletal forces spanning the nuclear envelope (NE) bring chromosomes close to each other to form the SC. The principle of such chromosomal motility is poorly understood but differs among organisms. The key components of the meiotic nuclear organization and chromosome dynamics during the pairing process, are proteins containing the SUN domain. Jointly with KASH, SUN-1 links the chromosomes through intact NE to the force-generating machinery in the cytoplasm, forming the LINC complex (Linker of Nucleoskeleton and Cytoskeleton). In *C. elegans*, the specific terminal part of chromosomes, called the paring center (PC) is located at the end of each chromosome. PCs are attached to the SUN/KASH domain, and they are essential for homologous pairing and synapsis [2–5]. HIM-8, ZIM-1, ZIM-2, and ZIM-3 from the family of zinc-finger proteins are responsible for this attachment to the SUN domain since they are able to bind both PC and SUN-1 [6,7]. Previously, it was demonstrated that PCs establish transient links to the cytoplasmic microtubule network [3,8,9], and this association remains dynamic during the leptotene and zygotene stages of meiotic prophase [6,7]. Disruption of tubulin (but not actin) polymerization abrogate the pairing and synapsis [3]. Pairing of homologous chromosomes is accomplished in the transition zone, where chromatin is polarized on the one side of the nucleus and appears crescent-shaped [10]. The pairing process is completed within 7–12-cell rows of the gonadal transition zone.

DEB-1 is the *C. elegans* orthologue of mammalian vinculin. Vinculin consists of a globular head, proline-rich neck region, and a rod-like tail domain that contains binding sites for many cytoplasmic proteins [11,12]. Vinculin was detected at the intercalated disks and costameres (heart-specific structure similar to the cell-matrix adhesions comparable to Z disk of the myofibrils) [13–16]. The customers share many components of focal adhesions (among others, integrins, talin, α-actinin, and focal adhesion kinase) [17].) DEB-1 has been known as an attachment protein found in dense bodies, connecting the actin filaments to basal sarcolemma in muscles [18]. Depletion of DEB-1 results in severe somatic/muscular disorders such as disorganized muscles and abolished pharyngeal pumping (PAT phenotype) [19]. In the worm gonad, this protein localizes to all somatic cells of the gonad, from the distal tip to the spermathecal, with a clear localization but unknown function in the distal gonad [20,21]. In the proximal gonad, DEB-1 is involved in generating mechanical forces in the myoepithelial sheath cells. These cells are specialized, smooth muscle-like cells, pushing oocytes into the spermatheca where they are fertilized. DEB-1 is directly linked to actin filaments generating gonadal sheath contraction. The effect of *deb-1* knock-down in germ cells was demonstrated by the appearance of endomitotic oocytes (the Emo phenotype) in the proximal ovary [22–24], hermaphrodite sterility [25] and production of dead eggs (RW3562 strain, described in *C. elegans* strain database of CGC University of Minnesota, USA).

Despite the localization of DEB-1 in the proximal part of the gonad [21] and DEB-1 depletion inducing decreased hermaphrodite fertility, so far there has been no evidence of DEB-1 involvement in meiotic progression. Here we show that DEB-1 has a specific role in the distal part of the hermaphrodite gonad, independent from ovulation activity of the contractile apparatus and its cytoplasmic function. We provide an evidence of DEB-1´s engagement in the meiotic prophase progression where the knock-down of *deb-1* affects attachment between homologous chromosomes which were paired and the SUN/KASH mechanistic module, and an initiation of synapsis during prophase I. In *deb-1* knock-down worms, the number of chromosomal univalents increases during diakinesis, jointly with the emergence of synapsis shaping defect and meiosis prolongation. Our study reveals an unexpected role of DEB-1 during critical moments of *C. elegans* gametogenesis, affecting chromosome dynamics and pairing.

## Materials and methods

### Strains and culture conditions

All strains were maintained and cultured under standard conditions at 20°C using *E. coli* OP50 as a food source, except when subjected to RNAi treatment [26]. We used these previously reported strains in this study (Table 1):

**Table 1.** List of *C. elegans* strains.

Name	Genotype	Origin
N2	wild isolate	Hodgkin J, Oxford University, Oxford, England
rrf-1	rrf-1 (pk1417) I	[65]
SUN1::GFP	sun1::EGFPII; sun-1(ok1218)yls34	Monique Zetka, MacGill University, Montreal, Canada
H2B::GFP/HIM-8::mCherry	him-8(tm611)IV; ttTi5605 vv Is17 II; unc-119(ed3)III; H2B::GFP vvIs17[pie-1p::mCherry::him-8::unc-54ter, unc-119(+)]	Monique Zetka, MacGill University, Montreal, Canada
CeLMN::GFP	baf-1::gfp::lmn-1 unc-119(+); [unc-119(ed3)]	Prof. Y. Gruenbaum, Alexander Silberman Institute of Life Sciences Hebrew University of Jerusalem

### Production of antibodies

The polyclonal antibodies *a*DEB-1/a*e and *a*DEB-1/b*f were produced by Clonestar Peptide Service (Brno, Czech Republic) using synthetic peptides of the following sequences: DEB-1/a*c: CRKLADRLNPQDR, and DEB-1/b*f: VSDHYDSSDEYD. Their localization is depicted in Figure S1 and specificity was tested on Western blot. The antibodies were designed to be exclusively specific for short (b*f) and long (a*e) isoforms (Figure S1). We showed that long isoform (a*e) are present only in the cytoplasm and not in the nucleoplasm and short isoform (b*f) are present in both the nucleoplasm as well as in the cytoplasm (Figure 2c). Whole-body lyzate, cytoplasmic or nuclear fractions were separated on SDS-PAGE and trans-blotted to the PVDF membrane. Membranes were incubated with the produced antibodies recognizing the correct band. Additionally, the specificity of the antibody was verified by a peptide blocking assay described below, prior to indirect immunofluorescence detection of both proteins in the fixed gonad. Sera with specific antibodies were pre-incubated with a specific peptide and used for immunofluorescence observation. Additionally, the signal reduction was monitored upon DEB-1 depletion, via interference with specific RNA against all DEB-1 isoforms. For all experiments, we used both rabbit sera rather than purified immunoglobulin.

### Protein lysates from C. elegans cultures

Cultures of synchronized N2 wild-type worms (further abbreviated as N2 (WT)) were prepared by seeding isolated embryos into ten 6 mm NGM plates, with OP50 bacteria feeding layer. After three days of incubation at 20°C adult worms were collected and transferred to 50 ml tubes by washing the plates with M9 buffer. The tubes were left on a rack for 15 min to allow the worms to pellet by gravity. Most of the M9 buffer was then removed and replaced with a one. In order to prepare cytoplasmic and nuclear extracts, we used *Q*proteome Nuclear Protein Kit (Qiagen). Lysates were loaded on 10% SDS-PAGE and separated by their size. The proteins were then trans-blotted to PVDF membrane using wet-tank blotting system. Membrane with proteins was blocked in 1% BSA in PBS-T for 1 h at room temperature. For detection of specific proteins we used the following antibodies: DEB-1/a*c rabbit polyclonal (diluted 1:100); DEB-1/b*f rabbit polyclonal (diluted 1:100); Tubulin rabbit polyclonal (Sigma Aldrich T5192, diluted 1:1000); H3 histone rabbit polyclonal (Sigma Aldrich H0164, diluted 1:1000), Lamin mouse monoclonal (abcam ab20396, diluted 1:500), SUN-1 rabbit polyclonal (ab103021, diluted 1:500). The membranes were incubated with the antibody for 1 h at room temperature. After subsequent repetitive washes in PBS, the membrane was incubated with goat-anti-rabbit 680 nm channel IRDye® secondary antibody and scanned by a near-infrared detection system (Odyssey LI-COR).

### RNA interference

For RNAi depletion of DEB-1, we prepared a construct with the full coding sequence of 3033 bp (ZC477.9). The *deb-1* sequence for RNAi was obtained by PCR from *C. elegans* cDNA using specific primers with ligation poly-linkers *Not*I and *Bam*HI and the product cloned to L4440 feeding vector (pPD129.36) [27]. The resulting construct was transformed into the HT115(DE3) RNaseIll-deficient *E. coli* strain. Transformed bacteria were spread on an NGM plate with ampicillin (50 mg/ml). Hermaphrodite worms at the L2 stage were transferred onto RNAi seeded plates and incubated for 48–72 h at 20°C. As a mock control we used an empty L4440 plasmid transformed in HT115 bacteria; otherwise the worms were incubated under the same conditions. The animals were immediately dissected for cytological analysis. For microinjection, we used RNA produced by T7 transcription (SP6/T7 Transcription Kit, Roche) from a plasmid with cloned in deb-1/a*c and deb-1/b*f. RNA obtained was purified (GeneJET RNA Cleanup and Concentration Micro Kit, Thermo Fisher) and microinjected into the middle part of the gonad. These were examined for progeny viability and brood size, and/or used for cytological analysis/examination, 48 hr after microinjection. Statistical differences were determined by Student’s t-tests.

### Cytological examination

Immunofluorescence staining was performed according to a standard protocol [28]. Briefly, the gonads were dissected in PBS and immediately fixed in 4% paraformaldehyde solution in PBS-T. Samples were snap-frozen and post-fixed in ice-cold methanol for 20 min, and blocked in 1% bovine serum albumin (BSA) in PBS-T. For immunodetection we used the following primary antibodies: DEB-1 mouse monoclonal (MH24, diluted 1:200; Developmental Studies Hybridoma Bank, University of Iowa, Iowa City, USA) [20]; SYP-1 guinea pig (diluted 1:800) [29]; HTP-3 guinea pig or rabbit polyclonal (diluted 1:200) [30]; PLK-2 rabbit polyclonal (diluted 1:200) [31], HIM-3 rabbit or chicken polyclonal (diluted 1:200) [32]. Dissected gonads were incubated with the primary antibody at laboratory temperature overnight. After washing with PBS, we used the following secondary antibodies: AlexaFluor 488 goat *anti-*mouse, *anti*-guinea pig and *anti*-rabbit (diluted 1:1000; Molecular Probes); AlexaFluor 555 goat *anti*-rabbit and *anti*-guinea pig (diluted 1:1000; Molecular Probes). Specimens were between microslide and coverslip in an *anti*-fading agent (VECTASHIELD; Vector Laboratories) with DAPI (1 mg/ml).

### Image acquisition and pairing analysis

Images obtained from the germ cells of the hermaphrodite gonad were acquired using Delta Vision Image Restoration System software (Applied Precission) at the nuclear equator, and 5 + 5 focal planes below and above it, at a separation of 0.2 μm. Maximal intensity projection (each representing 11 spanning 2 μm along the optical axis) were processed by soft-WoRX 3.0 software (Applied Precision) and deconvolved under standard conditions. For quantitative analysis of the PCs in the germ nuclei, we collected data from five control and 10 *deb-1*(RNAi) gonads divided into five evenly sized zones, from the first mitotic nuclei to late pachytene nuclei. We examined HIM-8 foci and scored the number of paired and unpaired foci. The HIM-8 signals were considered as paired/unpaired if the distance between their centers (identified as a maximum fluorescnence intensities in a line scan) was less than 0.7 μm [33]. Statistical significance was determined by Student’s t-tests.

### Live-cell imaging

For live-cell imaging of the meiotic nuclei, DEB-1 was depleted by feeding or microinjection 48 h ahead image acquisition. Worms were immobilized by Levamisol (25mM in water) and 8–10 worms at L4 stage were placed on *poly*-L-lysine coated slides. The dynamics of PC were captured by Delta Vision Image Restoration System (Applied Precision). Stacks of five optical sections separated by 0.25 μm were acquired every 5 s for 2 min, using x60/1.2 U Plan Apochromat objective (Olympus). Images were deconvolved by the Huygens software (Scientific Volume Imaging).

### Electron microscopy

Adult hermaphrodites (17–20 h post-L4) were subjected to high-pressure freezing. Freeze substitution and post-staining were carried out under standard conditions, as described elsewhere [34]. The 100 nm thick perpendicular sections were cut through the distal part of the hermaphrodite gonad. We detected DEB-1/b*f using rabbit polyclonal antibody (diluted 1:100), and subsequent incubation with donkey *anti*-rabbit IgG secondary antibody conjugated to 10nm gold particles (ab39597). Samples were observed on FEI Tecnai transmission electron microscopes. The XY coordinates of all gold particles are recorded and clustering or localization pattern of the labeling was quantified and statistically evaluated via GOLD – Software which is the stereological image analysis interpreting the density and distribution of gold particles [35].

## Results

### A new role of DEB-1 in C. elegans meiosis

**Figure 1.**
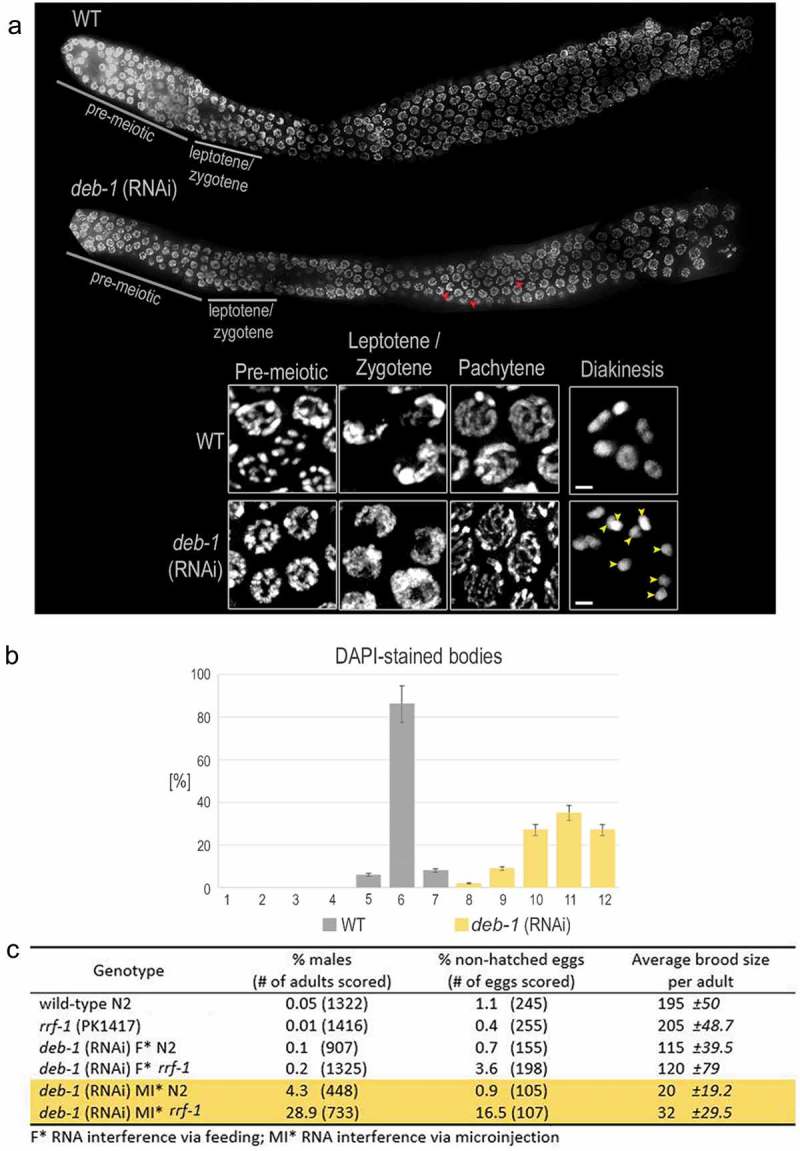
Effect of DEB-1 depletion on hermaphrodite gonad. a) Partial maximum intensity projection of distal part of DAPI-stained N2 (WT) hermaphrodite gonad, after 48 h of *deb-1* (RNAi) depletion. Meiotic nuclei (insets from different germlines) are present in relevant part of the gonad and appear to be comparable to the N2 (WT), except at diakinesis when chromosomal univalents are present (yellow arrowheads). ‘Transition zone-like nuclei’ are marked by a red arrowhead in pachytene stages. Scale bar, 1 μm. b) Statistical evaluation (histogram) of DAPI-stained bodies in N2 (WT) proximal gonad (n = 100), upon DEB-1 depletion (n = 100) [P = 0.005 compared to N2 (WT)]. c) Statistical evaluation of DEB-1 depletion via feeding and microinjection in the N2 (WT) and *rrf-1* worm strain. The following criteria were evaluated: incidence of males in the progeny, number of non-hatched eggs and their vitality, and brood size (the most prominent results are highlighted in yellow). All numbers were compared to untreated controls (N2 (WT) and *rrf-1*).

First, we investigated DEB-1 role via RNA interference [*deb-1* (RNAi)]. This was the method of choice as the well-established deb-1 mutants deb-1(st555) and deb-1(st554) are in the homozygous state paralyzed and they remain arrested in two-fold embryonic stage prior to hatch [36]. Obviously, for our study on meiosis, we need fully developed gonad typical for the L4 larval stage. The sequence of interfering RNA was homologous to the entire *deb-1* coding region and the reduction in DEB-1 expression protein was verified by the loss of immunofluorescence in excised gonads of N2 (WT) hermaphrodite worms. Moreover, the *deb-1* (RNAi) worms expectedly exhibited the Emo phenotype with no progeny [21]. The defect in the pumping rate in the proximal gonad results in worm sterility because eggs remain stuck in this part of the gonad, and are prevented to be laid. This phenotype makes it impossible to screen the effect of DEB-1 depletion in the progeny, e.g., to score the brood size, viability and the incidence of males. We thus used the *rrf-1(pk1417)* strain for RNA interference, as the mutants are resistant to RNA interference in the somatic gonad but sensitive in the germline as reviewed in [37]. Results from both N2 (WT) and *rrf-1* strains are presented (Figure 1c).

Upon DEB-1 depletion, proximal gonads were examined for their size and presence of meiotic stages, and compared to the N2 (WT) adult hermaphrodites. The latter develop gonads of normal size and organization as mitosis and meiosis progress (Figure 1a). In the transition zone, there is a partial chromatin polarization compared to the N2 (WT). This phenotype is caused by a decondensation of chromatin, manifested as a different pattern of DAPI staining (Figure 1a, inset). In N2 (WT), chromosomes then redispersed throughout the nuclear periphery upon entering pachytene (Figure 1a) while in DEB-1 depleted gonads, redispersion is only partial and ‘transition zone-like nuclei’ are apparent throughout all prophase I stages (Figure 1a, 3b, 5b, 6). During diakinesis, elevated number (8–12) of DAPI-stained compact bodies was observed (Figure 1a, inset, yellow arrowheads, and Figure 1b). A knock-down in N2 (WT) strain, by both feeding and microinjection, resulted in reduced brood size explicable by the observed Emo phenotype (Figure S5). The *deb-1* (RNAi) *rrf-1* strain showed reduced viable progeny compared to the untreated control. The brood size after DEB-1 depletion was reduced in average to 59 ± 22.2% in N2 (WT), and 58 ± 18.9% in *rrf-1* strain (RNA interference by feeding), and even more dramatically to 10.2 ± 5.8% in N2 (WT) and 15.6 ± 8.3% in *rrf-1* strain (RNA interference via microinjection). The numbers were compared to the mock-fed or microinjected control – N2 (WT) and *rrf-1* hermaphrodites. Importantly, laid but non-hatched eggs were observed only in *rrf-1 deb-1* (RNAi) worms both fed and microinjected but not in N2 (WT) *deb-1* (RNAi) worms, due to the Emo phenotype in N2 (WT). The reduction in egg vitality was most prominent in microinjected *rrf-1* worms (16.5% of eggs didn´t hatch, compared to mere 0.9% in the N2 (WT), Figure 1c). Moreover, depletion of DEB-1 in *rrf-1* resulted in a strikingly higher incidence of males (Him phenotype) in the progeny (up to 28.9%) compared to 0.05% in the N2 (WT) population.

In conclusion, the absence of DEB-1 results in the typical features of meiotic prophase defects such as increased number of univalents at diakinesis, high embryonic lethality and higher incidence of males in the progeny, despite the fact that the gonads are developed to the normal size and with the regular distribution of meiotic cells in the distal part of the gonad. Taken together, these results indicate a different role of DEB-1 in somatic cells versus the *C. elegans* meiosis where DEB-1 had so far an unknown role.

### DEB-1 isoform b*f localizes to early meiotic nuclei in C. elegans gonad

The DEB-1 protein is expressed in the entire worm body, in both embryos and mature hermaphrodites and the expression of DEB-1 jointly with other cytoskeletal proteins was detected in the proximal myoepithelial sheet [21]. To analyze and describe the dual role of DEB-1 in the distal gonad, we studied its localization in germ cells. With the help of originally produced polyclonal antibodies we identified two groups of long and short transcriptional isoforms of the *deb-1* gene (‘a’ to ‘h’, Fig. S1, 2c), varying in size (due to the alternative transcription and splicing) as well as in minor amino acid substitution in the coding regions (www.wormbase.org). The shorter DEB-1 isoforms b (369 aa) and f (358 aa) are homologous to the C terminus of four DEB-1 isoforms a/c/d/e (further denoted as a*e). Moreover, both of them contain the original exon (49 aa) at their N terminus. The g and h isoforms are produced by alternative splicing, are rather short (232 aa) and homologous with the N terminus of DEB-1 a*e. Their expression was confirmed by cDNA (based on RNAseq data). To produce distinct antibodies, we immunized rabbits with synthesized peptide fragments. We were able to produce a polyclonal antibody against the original N terminal extension, which made it possible to distinguish the b*f isoform (*a*DEB-1/b*f, 45 aa) from the other isoforms (Figure S1). In addition, we produced *a*DEB-1/a*e antibody against the fifth exon (710 aa) conserved in all four isoforms. This antibody yields the very same pattern (Figure S2) as the commercially available antibody MH24 (data not shown). In further experiments, we focused on the function of the short form of DEB-1 detected specifically by the DEB-1/b*f antibody.

We immunolocalized DEB-1 in the excised hermaphrodite gonad of untreated controls and *deb-1* (RNAi) worms (both N2 (WT) and *rrf-1*). As a positive control, we used MH24 monoclonal antibody against DEB-1 capable of detecting the epitope at the C terminus of the polypeptide, recognizing all eight known transcription isoforms (a*h) of DEB-1 [20]. The pattern of DEB-1 localization correlated with the known pattern, i.e. targeting DEB-1 within the muscle dense bodies and muscle-like fibers in the somatic gonad cells, as reported by Ono et al. [21] (Figure S3). Additionally, we observed a reproducible signal within the gonadal distal part, namely in the cytoplasm and the nuclear area of meiotic cells. This immunolocalization signal clearly disappeared upon RNAi depletion of DEB-1 (Figure S4). Jointly with findings on Emo oocytes, the results confirmed the depletion of DEB-1. On the other hand, the intense labeling of DEB-1 in the contractile apparatus was detectable even 48 h upon RNA interference (via feeding as well as via microinjection) explicable by a longer turnover of DEB-1 protein in myoepithelial cells. However, compered to the negative control, the proximal fibrils were disorderly organized (Figure S5).

**Figure 2.**
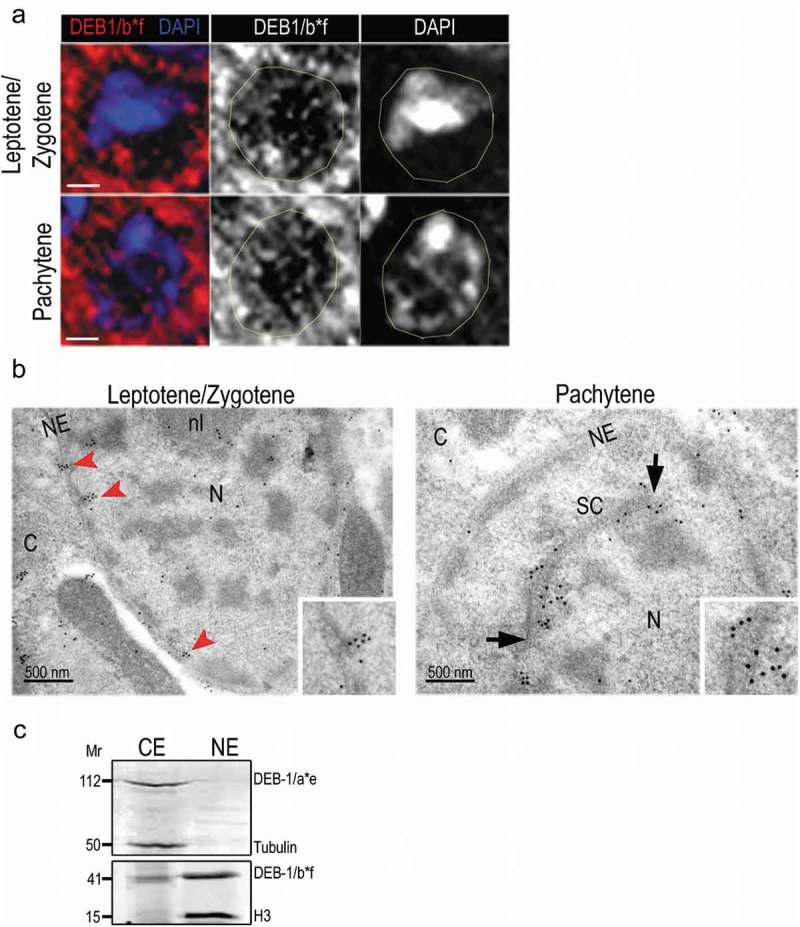
Localization of DEB-1/b*f in gonadal nuclei. a) Immunofluorescent localization of DEB-1 in the meiotic nuclei of N2 (WT) hermaphrodite gonad by structured illumination microscopy (N-SIM, maximal intensity projection of 1 μm z-stack). Scale bar, 1 μm. b) In leptotene/zygotene nuclei, DEB-1 is clustered in the close vicinity of the inner nuclear membrane (red arrowheads). During pachytene, DEB-1 lines the synaptonemal complex (SC, between black arrows). Transmission electron microscopy of ultrathin sections, scale bar, 500 nm. Used abbreviations: CP = cytoplasm, N = nucleus, NE = nuclear envelope, nl = nucleolus, SC = synaptonemal complex. c) Western blot of detected DEB-1´s long (DEB-1/a*e) and short (DEB-1/b*f) isoform in worm lysate, fractionated to cytoplasmic extract (CE) and nuclear extract (NE). To check fractions` purity we were also detecting cytoplasmic tubulin and nuclear histone H3.

Labeling of the gonad with an antibody against *a*DEB-1/a*e was comparable with the previously used MH24 monoclonal antibody (data not shown) [20]. The labeling pattern of DEB-1/a*e in the distal gonad was weak and diffused, with low intensity in meiotic cells. Identical to a previously reported MH24 pattern [21]. More interestingly, we identified strong and reproducible labeling in meiotic cells using a polyclonal antibody against DEB-1/b*f. After a detailed investigation of DEB-1/b*f isoform localization in the cytosol, we also investigated its nuclear localization (Figure 2a). To the best of author´s knowledge, this is the first observation of its kind. We used structured illumination microscopy (SIM; Nikon) with lateral resolution around 100 nm and identified DEB-1 inside the germ cell nucleus, partially colocalizing with DNA of the transition zone as well as pachytene nuclei (Figure 2a).

In parallel to immunofluorescent detection of DEB-1/b*f localization, we carried out immunogold labeling of DEB-1/b*f on N2 (WT) hermaphrodite whole body sections, to elucidate the observed nuclear localization also at ultrastructural level (Figure 2a). Specimens were prepared by minimally invasive method preserving the ultrastructure and antigens at the same time – high-pressure freezing and cryo-substitution, i.e. without any chemical fixation [34]. Transmission electron microscopy (TEM) analysis of DEB-1/b*f localization in germ-line nuclei confirmed localization to the early prophase I – in both leptotene/zygotene and pachytene stages. During leptotene/zygotene, DEB-1/b*f is localized in clusters in close vicinity of the inner nuclear membrane (Figure 2a, leptotene/zygotene, red arrowheads). Moreover, in the mid-pachytene regions of wild-type worms, DEB-1 localizes to the vicinity of the axial elements comprising the SC (Figure 2b, Pachytene, between black arrows).

To support this result a labeling density was calculated as a number of detected gold nanoparticles per area of the outlined region of interest in µm^2^. (Particles were detected, regions outlined and their area measured using Ellipse software (ViDiTo) with the „Gold“ plugin [35]). Resulting density for SC is 156.88, for N is 13.30 and for CP is 24. 03. Where CP stands for the cytoplasm and equals to the total area of cell section excluding the area of the nucleus, N represents the nucleus an equals to the area of the nucleus excluding the outlined area of the synaptonemal complex (SC). Presented values are the sum of particles and areas from all measured images. We also found DEB-1/b*f clusters in condensed chromatin. The presence of both DEB-1 isoforms was also tested in cytoplasmic and nuclear fractions in worm homogenates. We clearly demonstrate the presence of the long isoform exclusively in the cytoplasmic fraction whereas the short isoform was present in both of them (Figure 2c).

In summary, here we show, for the first time ever, the nuclear localization of the DEB-1 actin-binding protein in cells undergoing early meiosis in *C. elegans*. We used a polyclonal antibody against the original N-terminal extension to demonstrate that DEB-1 isoforms b and f localize to the meiotic cells and may play a dual role in gametogenesis.

### Homolog pairing of PC is established but not maintained in DEB-1 knock-down worms

**Figure 3.**
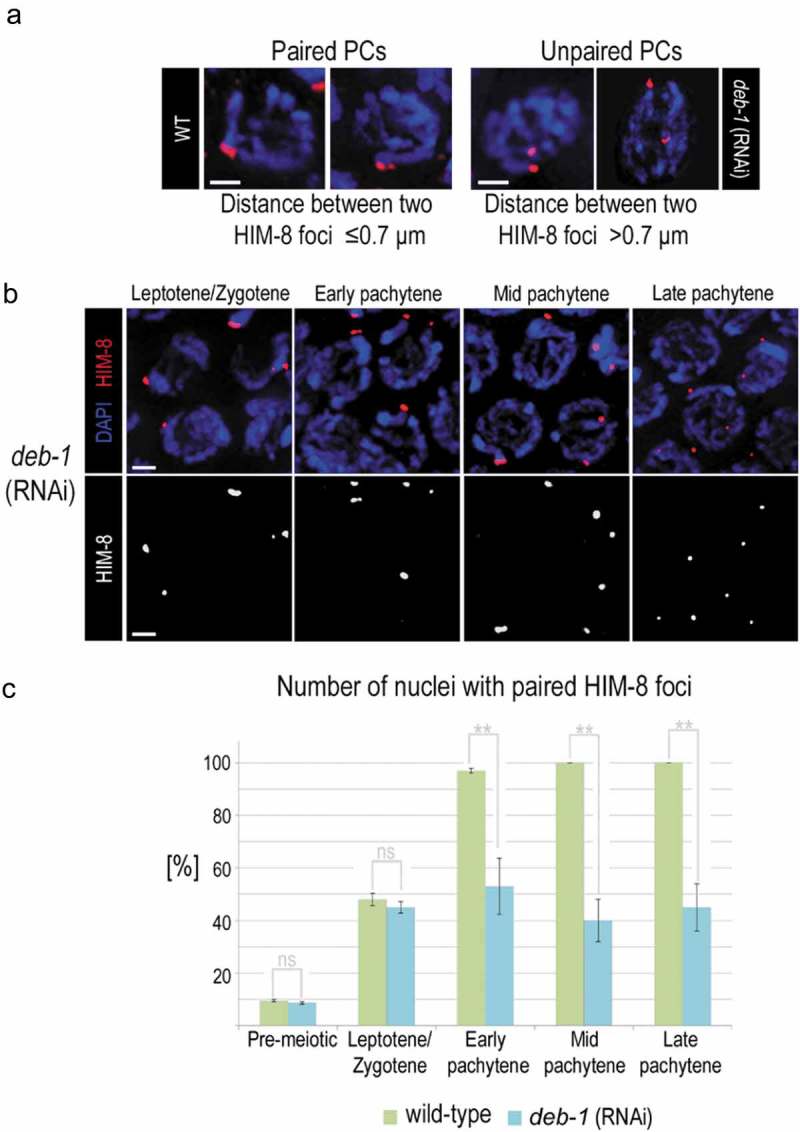
Chromosome pairing upon DEB-1 depletion. a) Immunofluorescence localization of HIM-8-labeled pairing centers of WT (non-treated control) and *deb-1* (RNAi) nuclei in pachytene, comparing paired and unpaired PCs. Scale bar, 1 μm. b) Localization of PCs in DEB-1 depleted gonad as prophase I is progressing. Scale bar, 1 μm. c) In the transition zone (letotene/zygotene) statistical evaluation of nuclei with paired and unpaired PC during prophase I showed tendency of PCs to aggregate but at pachytene, the number of paired nuclei in DEB-1 depleted animals started to decrease compared to untreated control (wild-type). ”ns” denotes significant difference [P ≥ 0.258]; two asterisks denote a very significant difference [P ≤ 0.01]. n values for wild-type are 41, 33, 31, 33, 34 nuclei and for *deb-1* (RNAi) 41, 59, 24, 55, 63 nuclei for pre-meiotic, leptotene/zygotene and early, mid, and late pachytene, respectively.

The localization of DEB-1 under the nuclear envelope (NE) in the early prophase led us to test the effect of DEB-1 depletion on the chromosome pairing center (PC) movement along the nuclear envelope, and the initiation of a homologous pairing process. This analysis was performed on nuclei containing X-specific HIM-8 foci in five equal-sized zones distributed along the distal-proximal axis of the germline. Five equal zones are composed by the first (most distal) part, containing exclusively pre-meiotic nuclei, followed by a transition zone containing nuclei in the leptotene/zygotene meiotic stage. The three subsequent zones represent a spanned meiotic prophase, from early through mid to late pachytene stage. If two individual HIM-8 foci centers (identified as a maximum fluorescnence intensities in a line scan) were separated in distance ≤0.7 μm (resp. >0.7 μm) were considered as paired (resp. non-paired) [33] and evaluated after DEB-1 depletion in both N2 (WT) and *rrf-1* strains (Figure 3a, N2 (WT) only).

The number of paired foci in the pre-meiotic and transition zones in *deb-1* (RNAi) worms was identical to N2 (WT) (47% ±1.2, control). However, with meiotic progression, we noticed a significant decrease of paired X chromosome pairing centers (HIM-8 PCs) (53% ±10.6) in *deb-1* (RNAi) worms, starting from early pachytene (Figure 3b). We measured the separation of pairing centers in fixed DEB-1-depleted gonads and found its increasing with the meiotic progression (Figure 3c). The distance in this gonad between detected non-paired chromosomes within the transition zone varied between 0.7 μm and 1.1 μm (0.9 μm on average), whereas during meiotic progression in mid and late pachytene it increased to 0.8–2.0 μm (1.4 μm on average) (Figure 3b).

As the localization of paring chromosomes in fixed gonad tells us little about the chromosome dynamics, we used a strain expressing HIM-8 fused to mCherry to analyze the motility of chromosomes upon DEB-1 depletion, and this allows us to track *in-vivo* the chromosomal movement.

**Figure 4.**
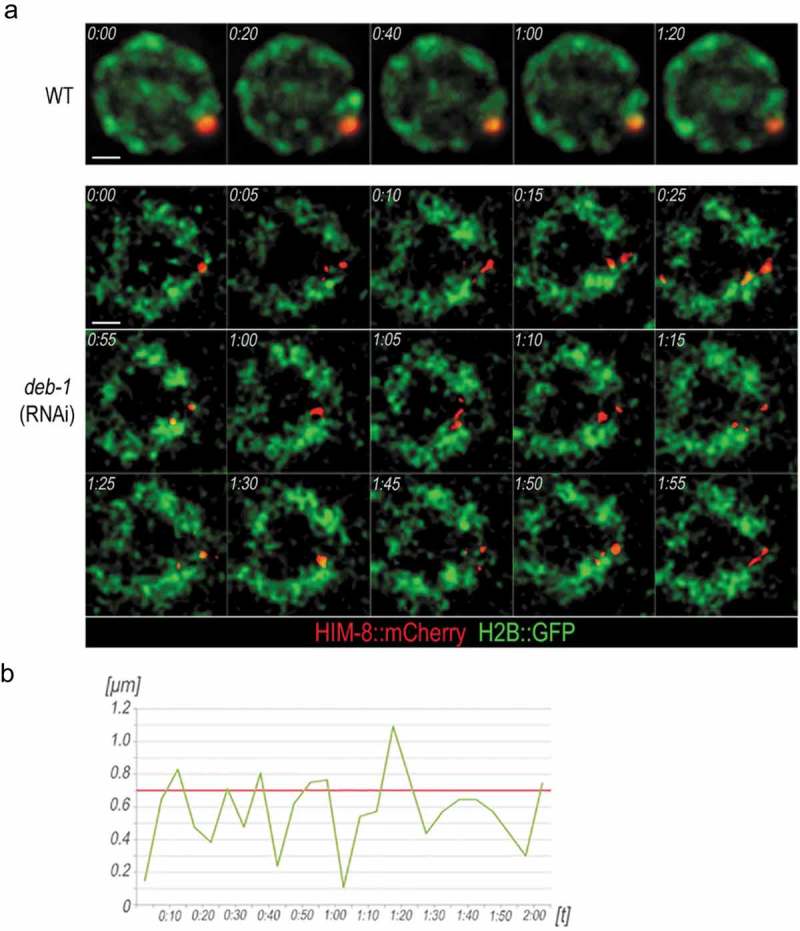
The HIM-8 pairing centers (PCs) remain dynamic in DEB-1 depleted worms. a) Time-lapse imaging of a single nucleus in wild-type and DEB-1 depleted worms in pachytene. DNA was visualized by H2B histone tagged with GFP (green), and PCs by HIM-8 tagged with mCherry (red). Scale bar, 1 μm. b) Evaluation of the distances between the PCs of pachytene nuclei after DEB-1 depletion (5s time steps). The range of fluctuating PCs was between 0.2 and 1.1 μm. Redline depicts the 0,7 μm threshold, under which the PCs were considered as paired.

Therefore, we used a strain H2B::GFP/HIM-8::mCherry (expressing HIM-8 fused to mCherry, DEB-1 wild-type) to analyze the motility of chromosomes upon DEB-1 depletion. We observed the dynamics of HIM-8 PCs during the pairing process and meiotic progression. In *deb-1* (RNAi) gonad, the X chromosome HIM-8 PC pairing was comparable in transition zone to the wild-type. However, as the pachytene stage progresses, HIM-8 PCs remain dynamic and repetitively separate and re-associate to form a single focus (Figure 4, Video S2). Statistical evaluation of HIM-8 PCs in *deb-1*(RNAi) gonad revealed a maximal separation of 1.1 μm. In contrast, HIM-8 PCs remain unseparated in the wild-type (Video S1).

It has been shown that repetitive actin polymerization and depolymerization is required for the movement of interphase chromosomes away from the nuclear envelope, after the transcription activation [38]. The pool of actin monomers plays an important role within the nuclear compartment. However, further investigation is needed to describe whether and how the mechanical forces are generated, transduced from the cytoplasm to the nucleus, and how they may affect the nuclear envelope (NE) and chromosomal regions linked to it via the SUN/KASH module [39–43]. Localization of DEB-1/b*f to the nuclear area (by immunofluorescence) and, more specifically, to the close vicinity of NE (by electron microscopy) during early meiosis when chromosomes undergo dramatic movement to pair with the homologs, prompted us to investigate the integrity and functionality of the SUN/KASH.

The PCs are elastic and capable of stretching over 1 μm during the pairing process in zygotene stage. These terminal parts of chromosomes can be separated for longer periods of time, prior to their association at early pachytene [9]. In *deb-1* (RNAi) worms, HIM-8 PCs are recruited to the nuclear envelope and initiate the pairing, as in wild-type. However, during prophase I progression, the pairing did not last, and HIM-8 PCs were found to detach and even recede from each other. We assume that upon DEB-1 depletion the cytoskeleton dynamics remained unaffected as we did observe chromosome movement and their recruitment to initiate the pairing process. While depolymerization of cytoskeleton leads to reduced chromosome dynamics [3,4,9] DEB-1 depletion has no such effect (Video S2). Thus, we suggest that the nuclear function of DEB-1 in meiocytes is independent of the cytoskeleton, which drives chromosome motility during their pairing.

As the stabilization of associated chromosome pairs was abrogated, we decided to investigate (1) the presence and functionality of the SUN/KASH mechanistic module ensuring chromosomes motility; (2) the presence and localization of synaptonemal proteins located between homologously recognized chromosomes; (3) the presence of crossing-over intermediates.

### Formation of SUN-1 aggregates lasts till late pachytene in DEB-1 knock-down gonads

The attachment of the chromosome ends to the nuclear envelope is dependent on the SUN domain-bearing inner NE proteins at the attachment sites. Absence of this interaction brings about random distribution of the PCs within the nucleus [5,44–46]. Thus, SUN-1 formed patches are highly dynamic and prerequisite of chromosome pairing [4]. Indeed, disruption of these integral NE proteins results in chromosome pairing defects during meiosis, as the PCs are randomly distributed within the nucleus. After homologous pairing, the chromosomes disperse in the nuclear space while still under the NE. At this point, SUN-1 spreads evenly on the nuclear membrane. Upon depleting of DEB-1, we observed juxtaposed but still highly dynamic chromosomes, thus we investigated NE topography in terms of SUN-1 distribution

**Figure 5.**
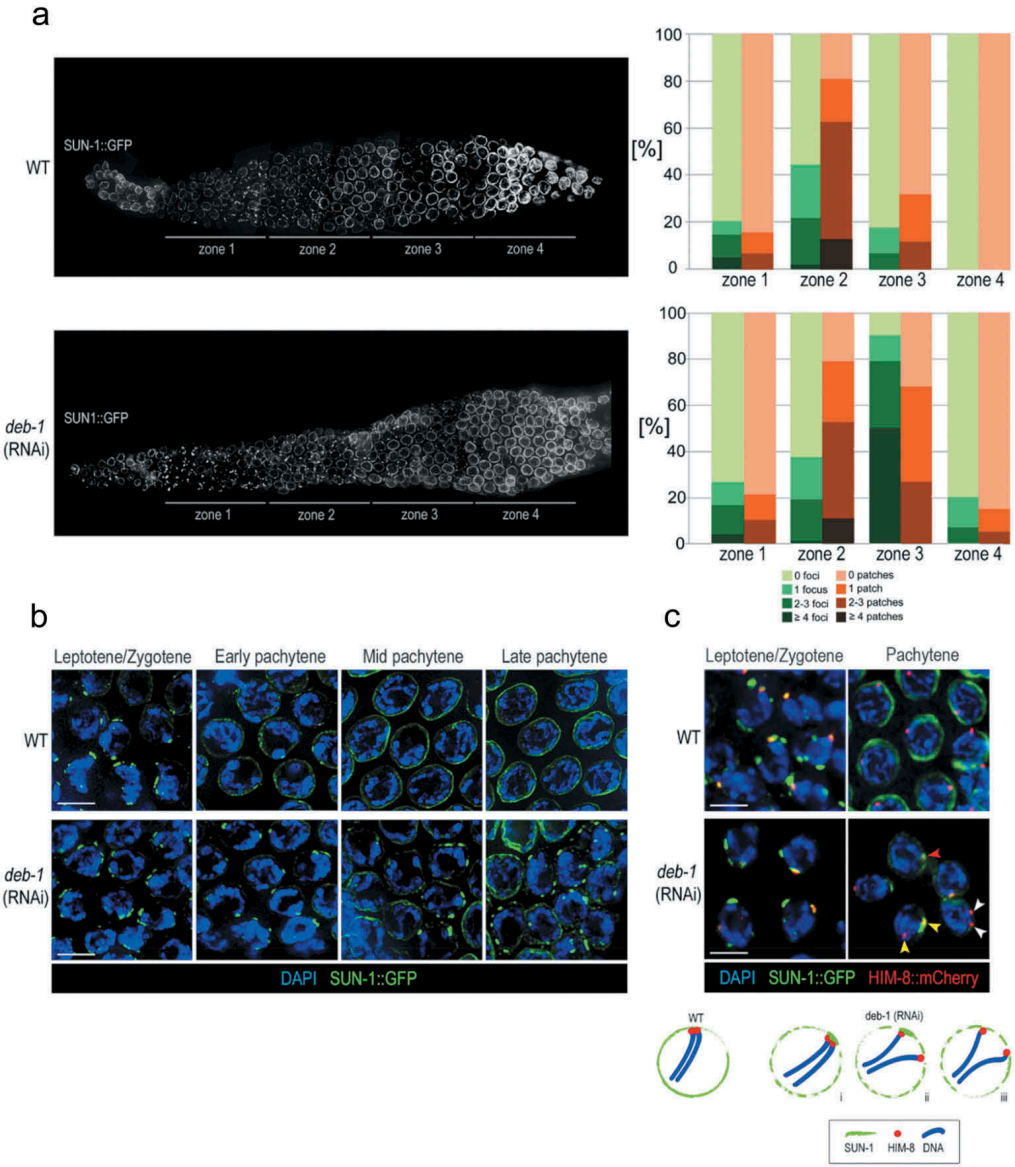
SUN-1 foci/patches persist till late pachytene upon DEB-1 depletion. a) Localization and statistical evaluation of SUN-1::GFP foci/patches present in the distal gonad of wild-type and *deb-1* (RNAi) worms (n = 89, 100, 95, 60 for WT nuclei (zone 1–5); n = 151, 113, 161, 50 for *deb-1* (RNAi) nuclei (zone 1–5). In WT worms the zone 2 nuclei displays the highest concentration of SUN-1 aggregates, whereas during zone 3–4, SUN-1 dissolves and localizes to the nuclear periphery. In *deb-1* (RNAi) worms the patches, as well as the foci, were still prominent until later zones. b) Detailed view on prophase I nuclei with apparent persisting SUN-1 foci upon DEB-1 depletion. Scale bar, 5 μm. c) Colocalization of HIM-8 PCs with the lasting SUN-1 foci/patches upon DEB-1 depletion. Scale bar, 1 μm. Scheme shows these encountered scenarios in pachytene: i) Paired PC localized to a lasting SUN-1 patch (red arrowhead); ii) Unpaired PCs and one PC localized to a lasting SUN-1 patch (yellow arrowheads); iii) Unpaired PCs and no PCs localized to the SUN-1 patches (white arrowheads).

Spherical SUN-1::GFP aggregates with a diameter ≤1.1 μm were classified as ‘foci’ while those larger in one dimension (>1.1 μm) were termed ‘patches’ [30]. In each gonadal zone, we counted the number and size of foci/patches associated with PCs per nucleus and the results are described in more detail in Figure 5a. The control hermaphrodite gonads expressing SUN-1::GFP (WT) in the transition zone and early pachytene featured both foci and patches. In the zone 1 of WT, approximately 80% of nuclei contained no patches or foci and around 20% of nuclei contain various numbers patches and foci. In the zone 2, the numbers of patches are increased (13% for 4 patches, 50% for 2–3 patches and 18% for 1 patch), similarly for the number of foci. In the zone 3, the most of the patches are dissolved and around 20% of nuclei still contained foci. In the zone 4 of WT, no foci and patches were detectable. Strikingly, in *deb-1* (RNAi) worms, while the zones 1 and 2 were similar to the WT, in the zone 3, over 70% (in total) of nuclei still contained 1–3 patches and over 80% contained a various number of foci. In the zone 4, the number of patches were strongly decreased (to around 17%) in most of the *deb-1* (RNAi) nuclei and we could still find 1–3 foci per nucleus in 20% of the nuclei (Figure 5a). This is in contrast with the zone 4 of WT nuclei where SUN-1 was evenly distributed at the NE.

Transgenic worm strains with mutated SUN-1 such as *sun-1(jf18)* (precluding its interaction with KASH) [4] or *plk-2(vv44)* (abrogated phosphorylation by PLK-2) [47] exhibit a phenotype with meiotic chromosomes that are unable to pair. These mutants develop gonads of normal size and organization, with missing transition zone and a defective attachment of chromosomes to SUN-1. Thus we asked if a depletion of DEB-1 affects the function of SUN-1, comparing our results with previously described mutants. However, our data clearly show that chromosome attachment to the SUN-1 remains unaffected. DEB-1 depleted gonads possess transition zone with lunar-shaped nuclei containing SUN-1 foci/patches comparable to WT. The SUN-1 aggregates in the pachytene nuclei in DEB-1 depleted gonad suggest an ongoing pairing process driven by the cytoskeletal machinery (Figure 5b). To confirm the attachment of chromosome to the SUN-1 foci in pachytene, we examined SUN-1 and HIM-8 colocalization in early prophase, in the nuclei of *deb-1* knock-down worms (Figure 5c). SUN-1 in the leptotene/zygotene formed patches and foci comparable to WT, with partially polarized nuclei and properly paired HIM-8 foci. On the other hand, during pachytene SUN-1 still formed patches/foci, but HIM-8 foci at the chromosomal terminus rarely colocalized with these dynamic trans-membranous modules. During early pachytene, around 30% (± 8%) of nuclei exhibited completely paired HIM-8 PCs (Figure 5c-i), comparable to the WT. Strikingly, with meiotic progression in pachytene, HIM-8 PCs were released from SUN-1 foci upon DEB-1 depletion (Figure 5c). We scored a number of nuclei with unpaired HIM-8 PCs (separated by >0.7 μm) and their association with SUN-1. In mid pachytene we observed around 68 ± 14% of nuclei with unpaired HIM-8 PCs; only one HIM-8 was still attached to the SUN-1 (Figure 5c-ii). Finally, in late pachytene, 80 ± 7% of nuclei exhibited unpaired HIM-8 PCs with disrupted SUN-1 colocalization upon DEB-1 depletion (Figure 5c – iii). SUN-1-positive nuclei (20 ± 3%) featured the same localization pattern as in the pachytene, with one HIM-8 focus colocalizing with the SUN-1 patch (Figure 5c).

In DEB-1-depleted worms, chromatin polarization and formation of SUN-1 aggregates associated with HIM-8 PC were observed during leptotene/zygotene. Both foci and patches were observed at the NE, jointly with clustered chromatin. SUN-1 patches appeared to be physically associated with HIM-8 PCs, mediating synapsis-independent pairing between homologous chromosomes, and probably acting as major sites of synapsis initiation [2].

These observations, jointly with earlier functional evidence [4], demonstrate a vital role of these NE components in meiotic chromosome dynamics. We found that upon DEB-1 depletion, the SUN-1/PC aggregates are present not only in the transition zone, but remain in a significant portion of nuclei until the late stages of prophase I (Figure 5b). Our results show that DEB-1 is important for completion of the chromosomal pairing process and thus for dissolution of SUN-1 aggregates during the pachytene stage. Therefore, we propose that DEB-1 could modulate the dynamics at chromosome ends by stabilizing the newly paired homologs.

### PLK-2 localizes to meiotic chromosome pairing centers in DEB-1 knock-down worms

During homologous pairing of chromosomes, an important role is also played by polo-like kinases (PLK), by facilitating the synapsis and PC-mediated inter-homolog interaction. Targeting of PLK to the nuclear envelope together with meiotic chromosomes enables a linkage between PC of homologous chromosomes with the cytoskeletal machinery and this linkage is needed for homology assessment [31]. In the next experiments, we examined the localization of PLK upon DEB-1 depletion, its colocalization with SUN-1 aggregates and PCs represented by HIM-8.

**Figure 6.**
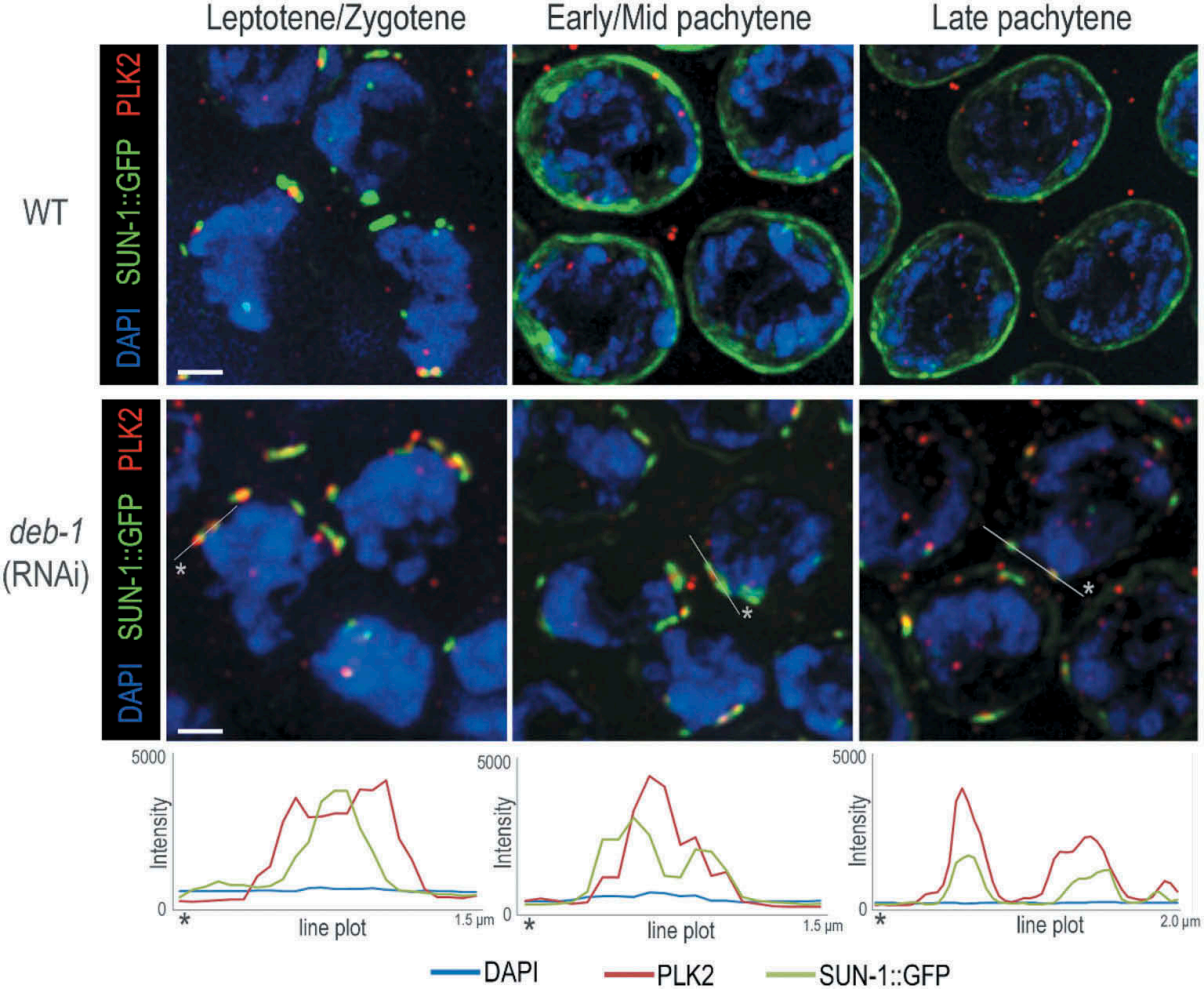
SUN-1::GFP colocalize with PLK2 upon DEB-1 depletion. Immunofluorescence localization of PLK2 in the meiocytes of wild-type and *deb-1* (RNAi) worms. Intensity plots demonstrate colocalization of both proteins in the transition zone (leptotene/zygotene) as well as at the pachytene stage for *deb-1* (RNAi). Scale bar, 1 μm.

Polo-like kinases (PLK-1 and PLK-2) target the pairing centers by ZIMs and HIM-8. Loss of these kinases inhibits chromosome pairing and licenses the synapses between non-homologous chromosomes. It has been shown that PLK-2 is required for PC-mediated inter-homolog interactions and meiosis-specific phosphorylation of SUN-1, and the establishment of dynamic SUN/KASH modules that promote homology pairing [31]. The discovery of these kinases provided a key insight into the regulation of homolog pairing and their targeting to the nuclear envelope, where meiotic chromosomes establish conserved linkages to cytoskeletal elements needed for homology assessment. Loss of PLK-2 function disrupts the formation of the NE bridge complex patches. In *deb-1* knock-down worms, we detected a clear colocalization of PLK-2 with SUN-1 foci in the transition zone, as well as in the pachytene where SUN-1 loci were remarkably preserved (Figure 6). This suggests that DEB-1 is active downstream of the SUN-1 activation. To conclude, SUN-1 focus/patch colocalized with PLK-2, but we hypothesize that the complex is insufficient to hold the PCs attached to the NE and initiate the homologous pairing. This implies DEB-1´s role in the mechanistic governing the early stages of prophase I.

### Depletion of DEB-1 results in chromosome pairing delay and synapsis defect

The depletion of DEB-1 affects the maintenance of homologously paired chromosomes. A fundamental issue arising from our results is the proper loading of the axial elements and SC components to the meiotic chromosomes upon DEB-1 depletion.

**Figure 7.**
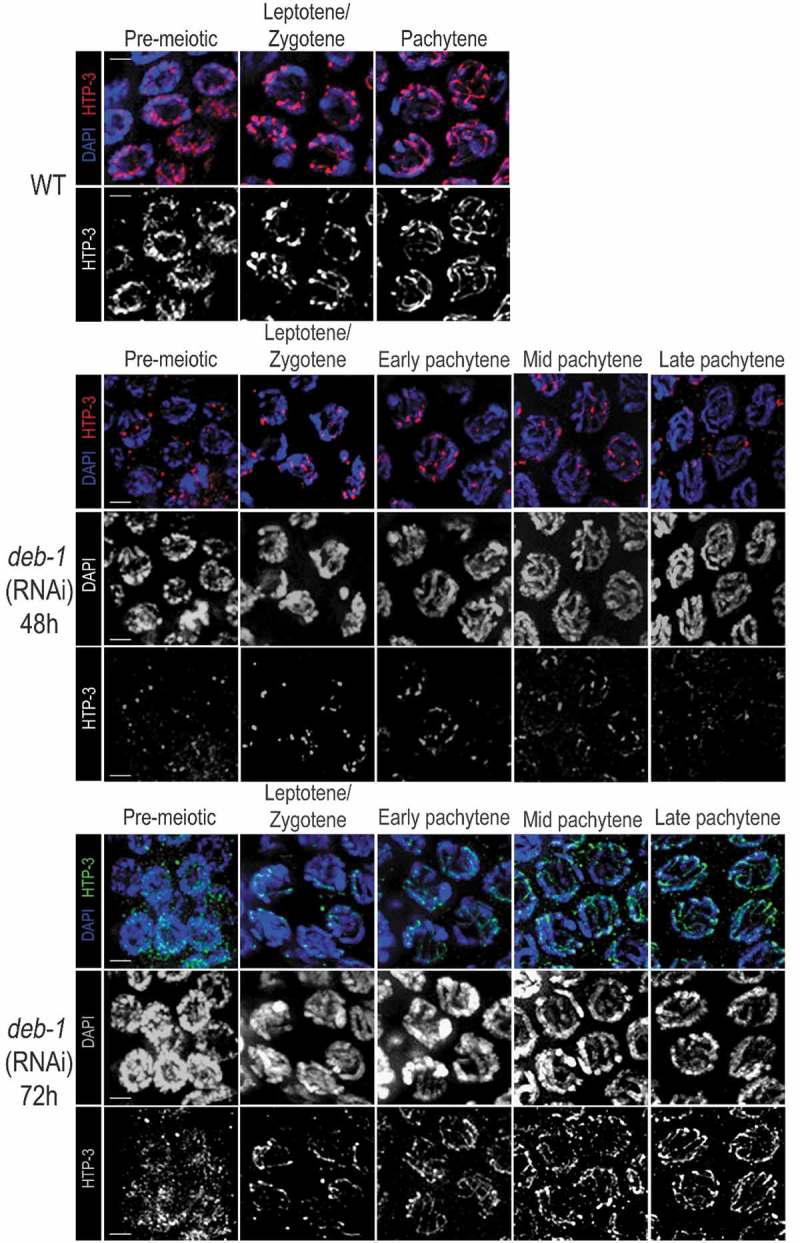
Altered localization of HTP-3 according to the time effect of *deb-1* (RNAi). HTP-3 pattern was mapped through the distal part of the gonad (pre-meiotic, leptotene/zygotene and pachytene – early, mid and late) in *deb-1* (RNAi) worms after 48h and 72h compared to the wild-type.

**Figure 8.**
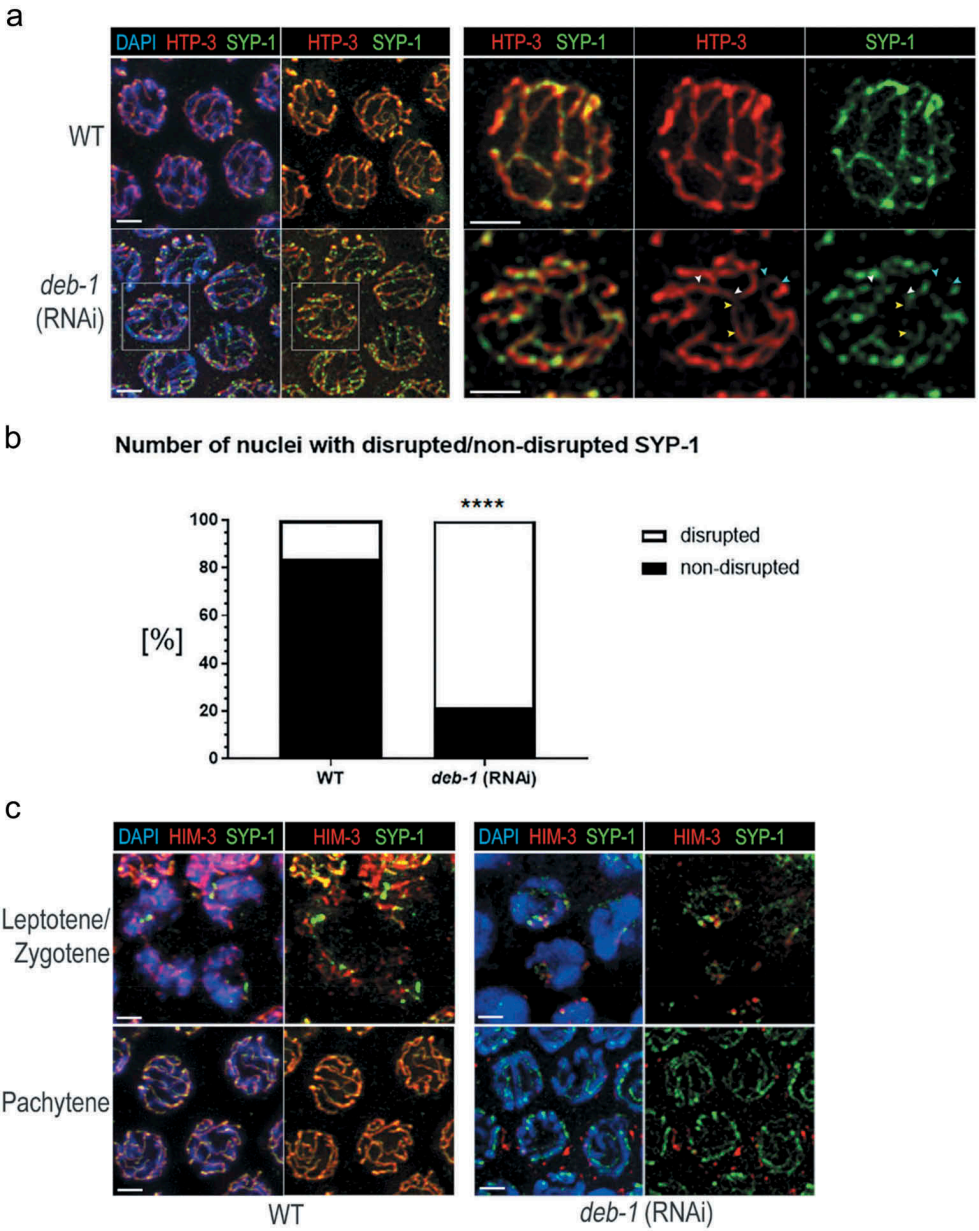
Depletion of DEB-1 results in synapsis defect. a) Immunofluorescence co-localization of the HTP-3 and SYP-1 protein. Upon *deb-1* (RNAi) depletion (72h), the fragmentation of HTP-3 is apparent, with disrupted loading of SYP-1. Scale bar, 1 μm. b) Statistical evaluation of disrupted/non-disrupted loading of SYP-1 for wild-type (n = 110 nuclei) and for *deb-1* (RNAi) (n = 103 nuceli). ”****” denotes significant difference [P ≤ 0.0001]. c) Immunofluorescence colocalization of HIM-3 and SYP-1 in the transition zone (leptotene/zygotene) and at pachytene. We observed only weak signal of HIM-3 and SYP-1 during the pairing process (leptotene/zygotene), and almost no HIM-3 proceed to the pachytene stage. SYP-1 forms short fragments following the DNA. Scale bar, 1 μm.

We investigated the localization of HTP-3, an axial component required for meiotic chromosome morphogenesis [48]. Immunolocalization of HTP-3 in *deb-1* knock-down worms revealed altered pattern of HTP-3 compared to the wild-type. Knock-down worms showed fragmented axial localization of HTP-3 in the pairing chromosomes depending on the time after RNAi treatment (Figure 7). Since the localization of HTP-3 after *deb-1* (RNAi) (48 h) was strongly disrupted (Figure 7), the presence of SYP-1 could not be observed at all. After *deb-1* (RNAi) (72 h), when the defect of DEB-1 depletion became weaker, we found that HTP-3 partially resembles the wild-type HTP-3 pattern, but still looks disrupted as well as the localization of the central element SYP-1 (Figure 8a, b). This observation suggests improper chromosome morphogenesis and pairing since both, the lateral and the central elements show defects in their assembly. This is in accordance with phenotypes observed in later stages (e.g. persisting of the SUN-1 aggregates, the presence of univalents in diakinesis) as well as the changes in the typical *C. elegans* gonadal nuclear reorganization (‘transition zone-like nuclei’ in pachytene). The absence of SYP-1 resulted in a failure to form chiasmata at diakinesis [49,50]. Chiasmata are structures resulted from cross-over recombination, and COSA-1 protein serves as a factor promoting the cross-overs. Thus, COSA-1 marks specifically the presumptive cross-over sites and concentrates to distinctive foci [51]. Therefore, we investigated COSA-1 pattern after DEB-1 depletion. Indeed, upon DEB-1 depletion we observed greater scatter in the number of COSA-1 foci (Figure S6). Taken together, these results indicate that the overall synapsis delay in *deb-1* (RNAi) worms leads to meiosis defects as the formation of a univalent and thus high incidence of males in the progeny and decreased brood size (Figure 1b-c). Despite the disturbed pattern of HTP-3 loading (most clearly pronounced shortly after RNAi treatment) and obvious pairing defects, the nuclei exhibited SYP-1 stretches, even if only short (Figure 8a). This can be explained by decreasing effects of RNAi treatment with the time. Additionally, we localized HIM-3 in the gonad of *deb-1* (RNAi) worms. Upon DEB-1 depletion, the HIM-3 pattern shows to be disrupted compared to the wild-type which resulted in the formation of a few patches in the nuclei of transition zone colocalizing with SYP-1 (Figure 8c). Almost no HIM-3 signal was observed when the nuclei proceeded to the pachytnene stage. The time-depending effect of RNAi treatment lead to the partial restoration of the HTP-3 and SYP-1 pattern 72 h after RNAi application (Figure 7). Similarly, the level of altered HIM-3 localization could depend on the time after RNAi treatment and later on, the HIM-3 loading to the SC can partially resemble the wild-type as we see a fractional SYP-1 assembly.

### DEB-1 interacts both with lamin and with SUN-1

**Figure 9.**
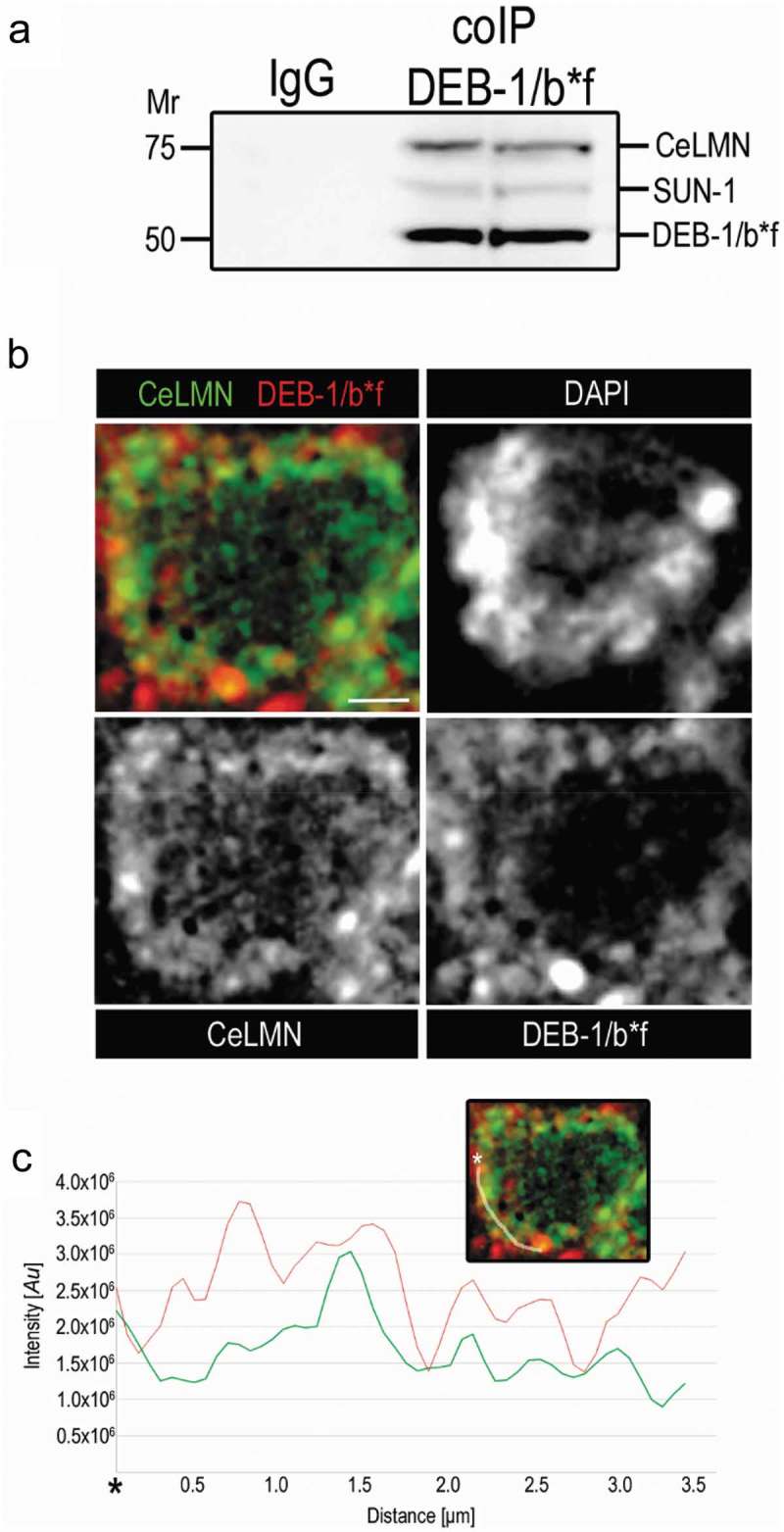
DEB-1 interacts both with lamin and with SUN-1. a) Co-immunoprecipitation of DEB-1/b*f from the nuclear extract of C. elegans. We detected CeLMN and SUN-1 interacting together with DEB-1/b*f. b) Immunodetection of DEB-1/b*f and its colocalization with CeLMN::GFP at the nuclear periphery of germ cell in the transition zone. c) Colocalization of these two molecules is apparent form the colocalization graph, measured along the line depicted on the figure above the graph. Scale bar, 1 μm.

To address the role of DEB-1/b*f at the nuclear periphery during first meiotic division, we decided to identify the interacting partners of DEB-1/b*f. Using co-immunoprecipitation we found that DEB-1/b*f interacts both with lamin (CeLMN) as well as with SUN-1, in nuclear extract from synchronized worm culture (Figure 9). Further, we colocalized DEB-1/b*f with CeLMN, tagged by GFP. From the microscopy pictures it is clear that DEB-1/b*f and CeLMN appear together at the nuclear periphery in the transition zone. Nevertheless, in contrast to results from co-immunoprecipitation, we did not detect colocalization of DEB-1/b*f with SUN-1 (data not shown). We can explain it by the dynamic and reversible interaction between these two molecules, but more experiments visualizing these two players *in vivo* need to be done, using super-resolution live cell imaging.

As we mentioned previously, SUN-1 together with KASH form the LINC complex that is a protein complex associated with both inner and outer membranes of the nucleus. SUN-1 associates with nuclear lamina at the inner nuclear membrane anchoring the LINC complex in the nucleus. However, there are still unidentified proteins accessorizing the LINC complex by anchoring SUN-1 to the lamina [52]. Our data strongly suggest DEB-1/b*f as a hot candidate to be the anchor, constituting the interconnection between highly dynamic SUN-1 and the rigid lamina.

## Discussion

### The role of DEB-1 in chromosome dynamics and homologous interaction

Here we show a novel nuclear role for DEB-1, additional to its´ already described independent function in somatic cells in *C. elegans* gonads dedicated to the actin-binding ability. The fundamental question addressed in our experiments was how DEB-1 is involved in early meiotic progress; the impact on the chromosome interaction with cytoskeleton, dynamics, and coordination of pairing, and formation of SC between homologous chromosomes. We bring novel data showing a novel actin-binding protein localized to the nuclear space of early prophase meiotic cells of *C. elegans*. DEB-1 is located to the nuclear membrane at the leptotene/zygotene stage, and it may seem that DEB-1 decorates also the SCs that are being formed during pachytene. Depletion of DEB-1 resulted in a phenotype showing from a dramatically reduced progeny and high incidence of males, thus indicating defective meiotic prophase. Indeed, while HIM-8 PCs were aggregating normally they were unable to stabilize the homologous pair and undergo subsequent homolog pairing. Monitoring of the distance measured between individual HIM-8 PCs showed their increased movement during the pairing process The pairing centers in the transition zone fluctuate between 0.2 and 2.0 μm, same as during the normal process of pairing in early prophase. Although HIM-8 pairing centers were correctly linked to the transmembrane SUN/KASH machinery, thus transducing the cytoskeletal forces, this situation lasted until late pachytene. Such an observation contrasts with scattered chromosomes during pachytene in the nuclei of wild-type hermaphrodites [30]. When DEB-1 was depleted, chromosomes failed to synapse, despite correct homologous pairing among PCs (tested on X chromosomes). We assume that the same or similar situation can be deduced for the PCs of autosomes since we see 8–12 DAPI-stained bodies (Figure 1a, b) which suggests that the synapsis of autosomes might be also disrupted. We observed altered loading of lateral elements (HTP-3, HIM-3) between juxtaposed PCs, and defective loading of SC elements (SYP-1) in pachynema. Taken together, these data indicate that DEB-1 is a novel nuclear factor in meiotic prophase I, acting independently of the cytoskeleton, and affecting the homologous pairing process and synapsis coordination. The DEB-1 activity is unusual in the context of known chromosome-nuclear interaction, and raise questions as to how the homologous pairing is in reality accomplished during meiosis, and how this novel factor fits into the already described mechanisms regulating the selective formation of homolog synapse.

Our results suggest DEB-1´s role in immobilizing the PCs and/or providing a counterforce to the dramatic cytoskeletal movement. Germ cell nuclei are embedded in a dense meshwork of microtubule bundles linked to the mechanistic module through which forces are exerted on chromosomes [53]. The DEB-1 protein interacts with actin filaments jointly with UNC-52/perlecan and PAT-3/integrin, and these are predominantly present in the cytoplasm and near the membrane of the muscle dense body [18,19,45]. The relationship between microtubules and the contact sites of cytoskeletal actin and DEB-1 plays an essential role in microtubule capture, stabilization against depolymerization, and possible nucleation of the microtubule assembly [54–56]. However, we have not observed any disruption of the integrity of germ cells or scaffolding during the meiotic stages in *the rrf-1* hermaphrodite gonad, as proved by localization of MOE::GFP upon DEB-1 depletion (data not shown). Previously, it has been reported that microtubules rather than actin, are the mediators of homolog pairing and synapsis in the early prophase in *C. elegans* [3,9]. Here we report that upon DEB-1 depletion, chromosomes remain dynamic and normally initiate the pairing process, in contrast to chromosome scaffolding and immobilization upon cytoskeleton destabilization [9]. We suggest that the nuclear role of DEB-1 is independent from cytoskeletal polymerization, as DEB-1 depletion did not disrupt the cytoskeleton attachment to the SUN/KASH module. The process of chromosome pairing is rather complex and still awaits elucidation. It has been known that PCs are attached to their partners mediating the attachment to the cytoskeleton through molecular motors, but some factors still remain to be identified [53]. We propose the DEB-1 protein as a new player localized to the nuclear membrane, and involved in chromosome pairing by assisting the cytoskeletal machinery in homolog juxtaposition, stabilization, and initiation of the synapsis.

It has been reported that homologous pairing is mediated by attachment of PCs (located close to one end of each chromosome) to the inner nuclear envelope [6,7] and the microtubule cytoskeleton [3,9]. The earliest stages of the pairing process are likely to involve interactions of the pairing centers and homologous interstitial regions through a recombination-independent process as reviewed in [57–59]. In most organisms, these heterochromatic regions appear to dynamically cluster and de-cluster during the early prophase, suggesting their important role as reviewed in [60]. Our data suggest that DEB-1 acts as an additional and independent regulatory factor facilitating chromosome attachment to the NE via PCs, and their active motion during the pairing process, thus probably stabilizing the juxtaposed homologous chromosomes, and initiate the pairing process.

Previous studies have indicated that once initiated, SC formation in *C. elegans* is highly progressive and insensitive to homology [3]. Moreover, based on the circumstantial evidence that PCs act as major sites of synapsis initiation [2], it is plausible that the depletion of DEB-1 is specifically affecting mechanisms linked to these sites. This is consistent with no or weak loading of lateral and central elements of SCs upon DEB-1 depletion even in situations when PCs were homologously associated.

We believe our data help to clarify the pairing and synapsis initiation mechanisms at the NE. However, the nature of the affinity between homologous chromosomes that underlies the cell´s ability to differentiate between homologous and non-homologous interactions remains unknown. However, the mechanisms responsible for mechanical stabilization of associated homologous (but not non-homologous) PCs do exist, and the physically limit the accessibility of the chromosome´s terminal parts [61–63]. This will prolong the chromosome juxtaposition arrest required for loading of SC factors initiated by the homologous pairing of PCs. Immunogold localization of DEB-1 at an ultrastructural level has revealed clusters under the NE, but also its localization along the newly formed SC. It is possible that not only PCs themselves, but also the extended axes of the already coupled parts chromosome pairs undergo this stabilization. PCs have been shown to be essential for accurate meiotic segregation, but not necessarily required for homolog pairing and synapsis [2,64]. In *C. elegans*, homologous pairing and synapsis can be fully uncoupled from recombination, and thus no kind of homologous chromosome stabilization has been reported so far. Thus, a clear evidence of DEB-1´s involvement in mechanical stabilization of homologs prior to loading of lateral and central elements and SC extension is yet to be provided.

The specific mechanisms underlying chromosome motility vary among organisms but the outcome is similar in all cases: vigorous back-and-forth movement (as fast as 1 μm/sec in budding yeasts) led by chromosome ends and directed by cytoskeletal components via a direct link through the NE [53]. The exact role of these movements remains unknown although published data indicate they present a stringency factor, by eliminating unwanted inter-chromosomal associations or entanglements accompanying the process of homologous pairing [53].

### DEB-1 as a novel component of the LINC complex

The appropriateness of DEB-1 to be a good candidate for the anchoring molecule in the LINC complex is supported by properties, which are known from its mammalian orthologue vinculin, such as: i) its structure able to form active and passive auto-inhibitory conformation; ii) numerous interactors like kinases, proteins, and lipids; iii) its ability to activate the pathways downstream from extracellular stimuli. Unless these were described on the site of the cytoplasmic membrane, there are some indicators suggesting that the function can be similar resp. adapted to the events under the nuclear membrane. Vinculin is an adapter protein with binding sites for more than 15 proteins. Biochemical and structural analyses have contributed to a detailed knowledge about the potential binding partners and the understanding of how their binding may be regulated. Vinculin is composed of head, neck and tail domains with the interaction between head and tail domains masking the binding sites for vinculin-binding partners [65]. Chen et al. [66] were able to demonstrate that vinculin undergoes conformational changes when localizing to focal adhesions. They showed that only the active extended form of vinculin localizes to active sites on the cytoplasmic membrane whereas the folded inactive form resides in the cytoplasm. It is thought that vinculin exists in an equilibrium between active and inactive states and it can be stabilized in the active form by interactions with a subset of binding partners [11,66–68]. With its prominent links to the actin, vinculin is a major candidate for mediating transmission of signals between cell compartments [69,70].

This poses a question as to where exactly does DEB-1 actually takes part in the regulatory process of transducing cytoskeletal forces to the nucleus interior via the SUN/KASH complex bridging the nuclear envelope. Being consistent with the scheme describing interphase cells and the association of SUN-1 with the outer nuclear membrane, our data identify the missing link in terms of DEB-1 activity, affecting chromosome movements. In early meiotic prophase, NE proteins aggregate into patches at the nuclear periphery. In *C. elegans*, chromosomal PCs bind members of the ZIM family such as HIM-8 and link up (via the inner NE protein SUN-1) to microtubule-motor proteins in the cytoplasm [3,4,71]. The laboratories of Dernburg and Zetka reported that aggregation of PCs at the nuclear periphery is facilitated by the polo kinase PLK-2 and, to a smaller extent by PLK-1 [31,72]. Both laboratories have shown that a loss of the *plk-2* allele delayed or prevented pairing and synapsis formation. PLK-1 and PLK-2 are thus instrumental in the pairing of homologous chromosomes at the NE. In *deb-1* knock-down worms, both the localization of PLK-2 and its link to the nuclear patches was preserved during leptotene/zygotene. Moreover, SUN-1 phosphorylation may directly affect its ability to self-associate or to bind other proteins that affect the formation of higher-order SUN-1-containing complexes, such as B-type lamin in *C. elegans* [73]. Given the fact that i) highly-motile SUN-1 foci are link to the patches during early prophase but unable to stabilize these aggregates, and ii) DEB-1 co-immunoprecipitated with lamin and SUN-1, DEB-1 is likely to be an important new player in LINC complex.

**Figure 10.**
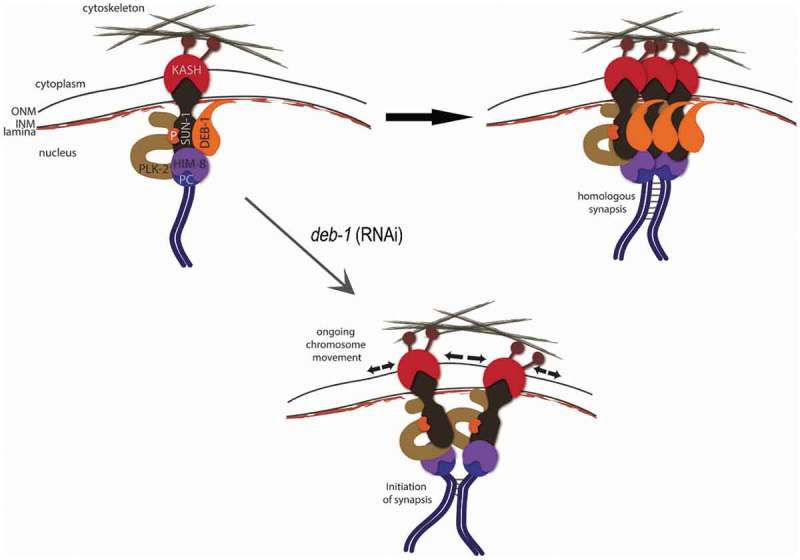
DEB-1 as a novel component of LINC complex. At nuclear periphery, SUN-1 protein at the inner nuclear membrane (INM) forms a trans-membranous complex jointly with the KASH protein on the outer nuclear membrane (ONM). The complex is linked to the cytoskeleton via dynein in the cytoplasm, and recruits the terminal pairing centers (PC) in the nucleus, where it promotes pairing and synapsis of homologous chromosomes. DEB-1 is likely to play anchoring role between SUN-1 and lamina. Thus, its depletion abolishes the stabilization of SUN-1 aggregates, keeping them in highly dynamic state. This results in defects/delays in loading of SC components.

Jespersen and Hawley [74] and a few others [52,75,76] suggested a comprehensive scheme for the recruitment of several factors to the nuclear envelope during pairing and synapsis formation. Interestingly, the authors speculated about an unknown ‘factor X’ localized to the nuclear periphery in the pairing process, close to both SUN-1 and to lamina [74]. Here we provide evidence that the localization and function of DEB-1 is equal to factor ‘X’ (Figure 10). However, several important issues remain to be elucidated, such as how is DEB-1 localized/transported to the nuclear periphery, and which part of the molecule is responsible for a direct or indirect interaction with one of the LINC complex components during meiosis. Future studies aimed at identifying DEB-1 binding partners in the worm will help to address this issue. Nevertheless, even our current data on chromosome dynamic in meiotic prophase are consistent with the scheme considering data of the current studies dealing with (Figure 10, reprinted from [74]).
