# Supplementary material for: Pairing of homologous chromosomes in C. elegans meiosis requires DEB-1 - an orthologue of mammalian vinculin
Source: Nucleus. 2019 May 8;10(1):93–115. doi: 10.1080/19491034.2019.1602337 (PMC6527391; doi:10.1080/19491034.2019.1602337)
Supplement: Supplemental Material [file kncl-10-01-1602337-s001.pptx]

## Slide 1
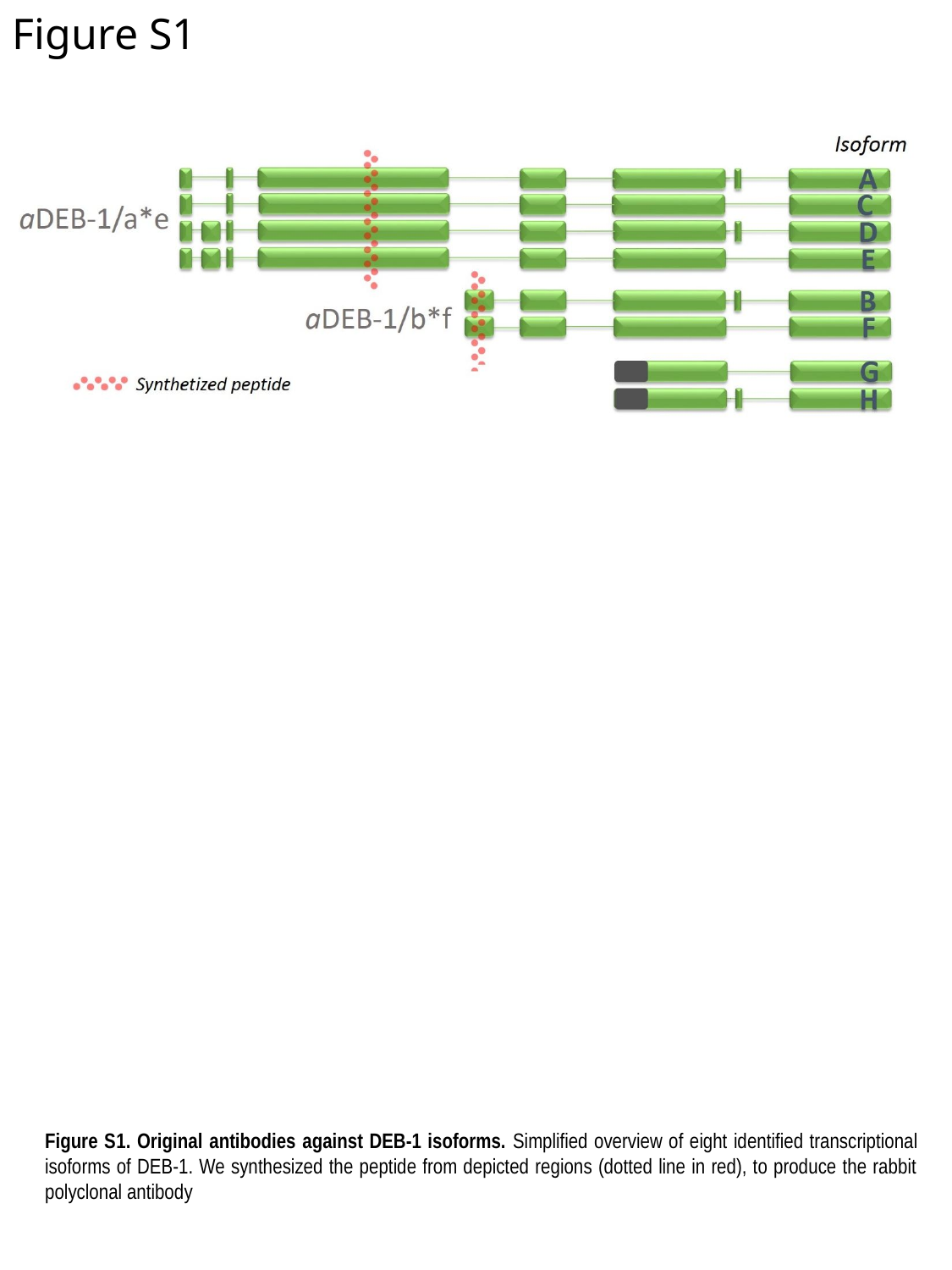

Figure S1
Figure S1. Original antibodies against DEB-1 isoforms. Simplified overview of eight identified transcriptional isoforms of DEB-1. We synthesized the peptide from depicted regions (dotted line in red), to produce the rabbit polyclonal antibody

## Slide 2
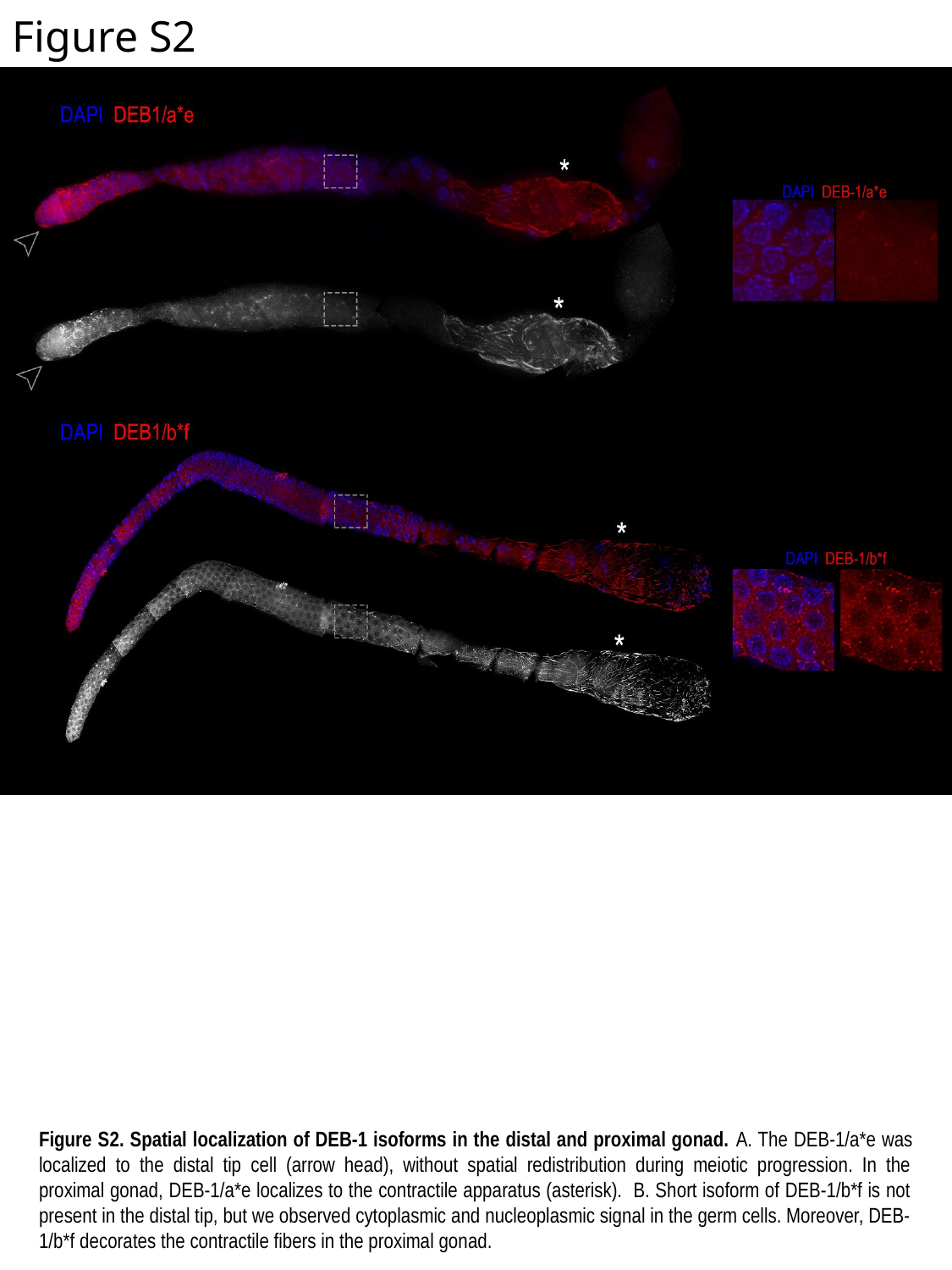

Figure S2
A
Figure S2. Spatial localization of DEB-1 isoforms in the distal and proximal gonad. A. The DEB-1/a*e was localized to the distal tip cell (arrow head), without spatial redistribution during meiotic progression. In the proximal gonad, DEB-1/a*e localizes to the contractile apparatus (asterisk). B. Short isoform of DEB-1/b*f is not present in the distal tip, but we observed cytoplasmic and nucleoplasmic signal in the germ cells. Moreover, DEB-1/b*f decorates the contractile fibers in the proximal gonad.

## Slide 3
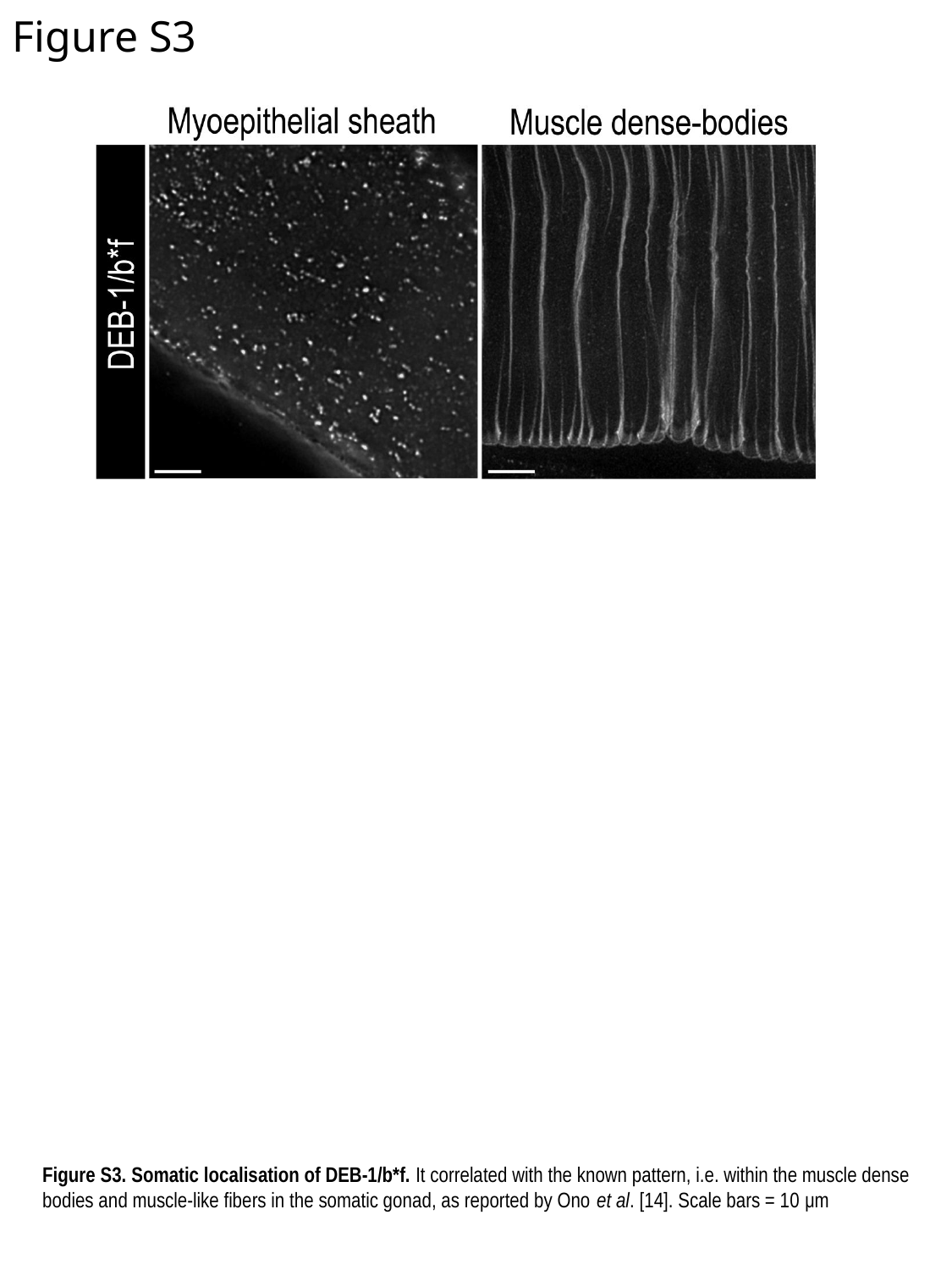

Figure S3
Figure S3. Somatic localisation of DEB-1/b*f. It correlated with the known pattern, i.e. within the muscle dense bodies and muscle-like fibers in the somatic gonad, as reported by Ono et al. [14]. Scale bars = 10 μm

## Slide 4
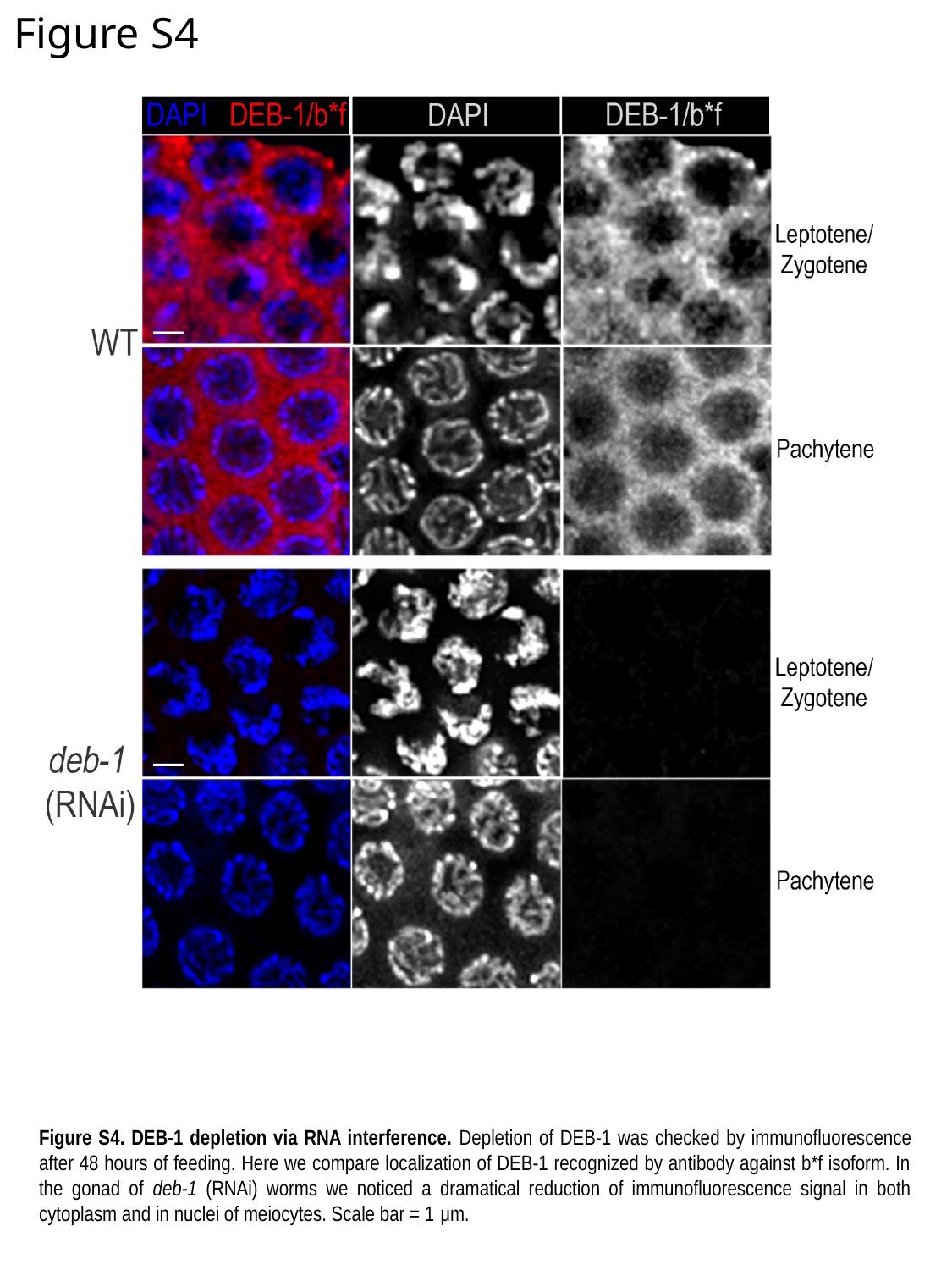

Figure S4
Figure S4. DEB-1 depletion via RNA interference. Depletion of DEB-1 was checked by immunofluorescence after 48 hours of feeding. Here we compare localization of DEB-1 recognized by antibody against b*f isoform. In the gonad of deb-1 (RNAi) worms we noticed a dramatical reduction of immunofluorescence signal in both cytoplasm and in nuclei of meiocytes. Scale bar = 1 μm.

## Slide 5
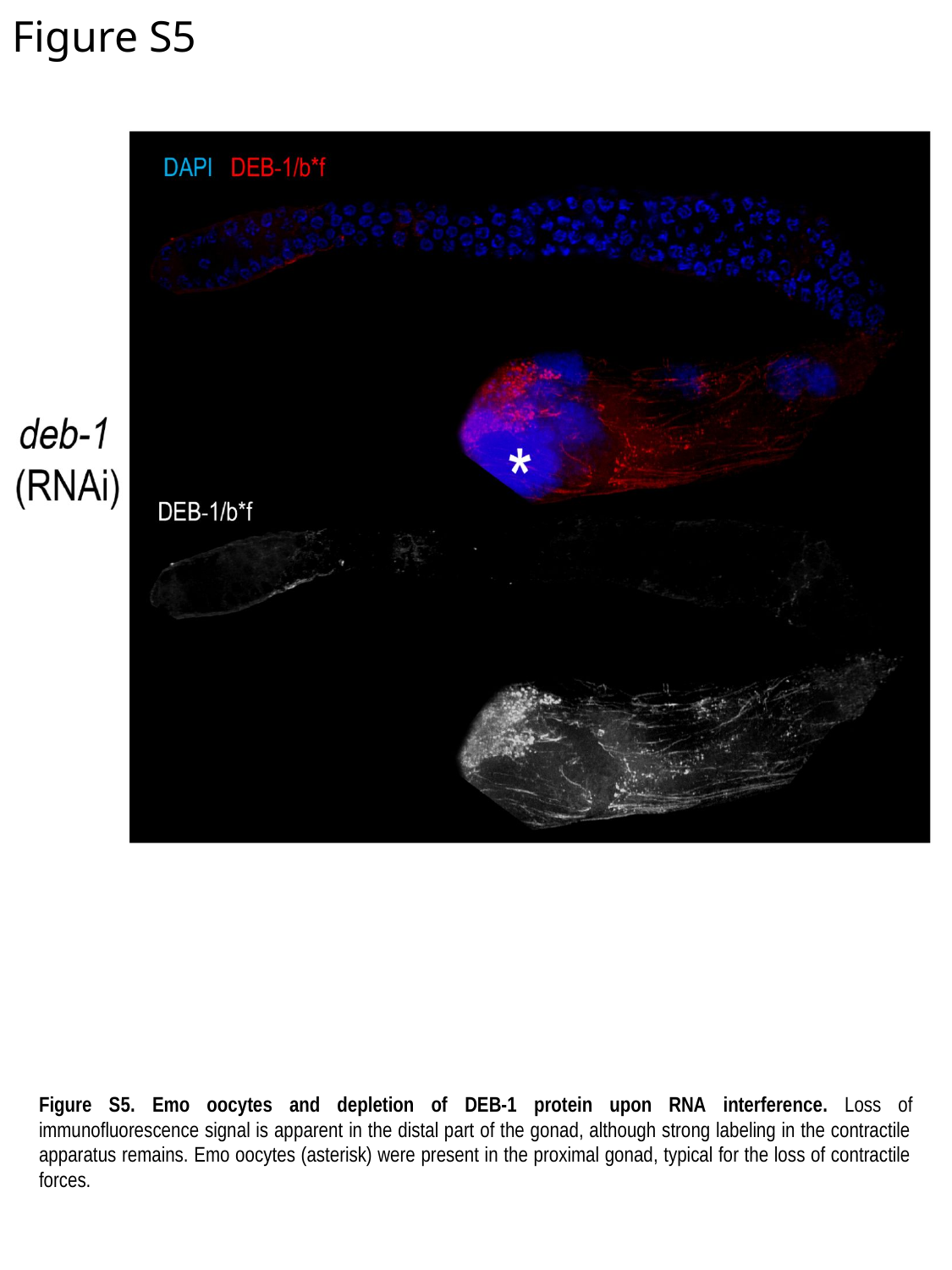

Figure S5
A
Figure S5. Emo oocytes and depletion of DEB-1 protein upon RNA interference. Loss of immunofluorescence signal is apparent in the distal part of the gonad, although strong labeling in the contractile apparatus remains. Emo oocytes (asterisk) were present in the proximal gonad, typical for the loss of contractile forces.

## Slide 6
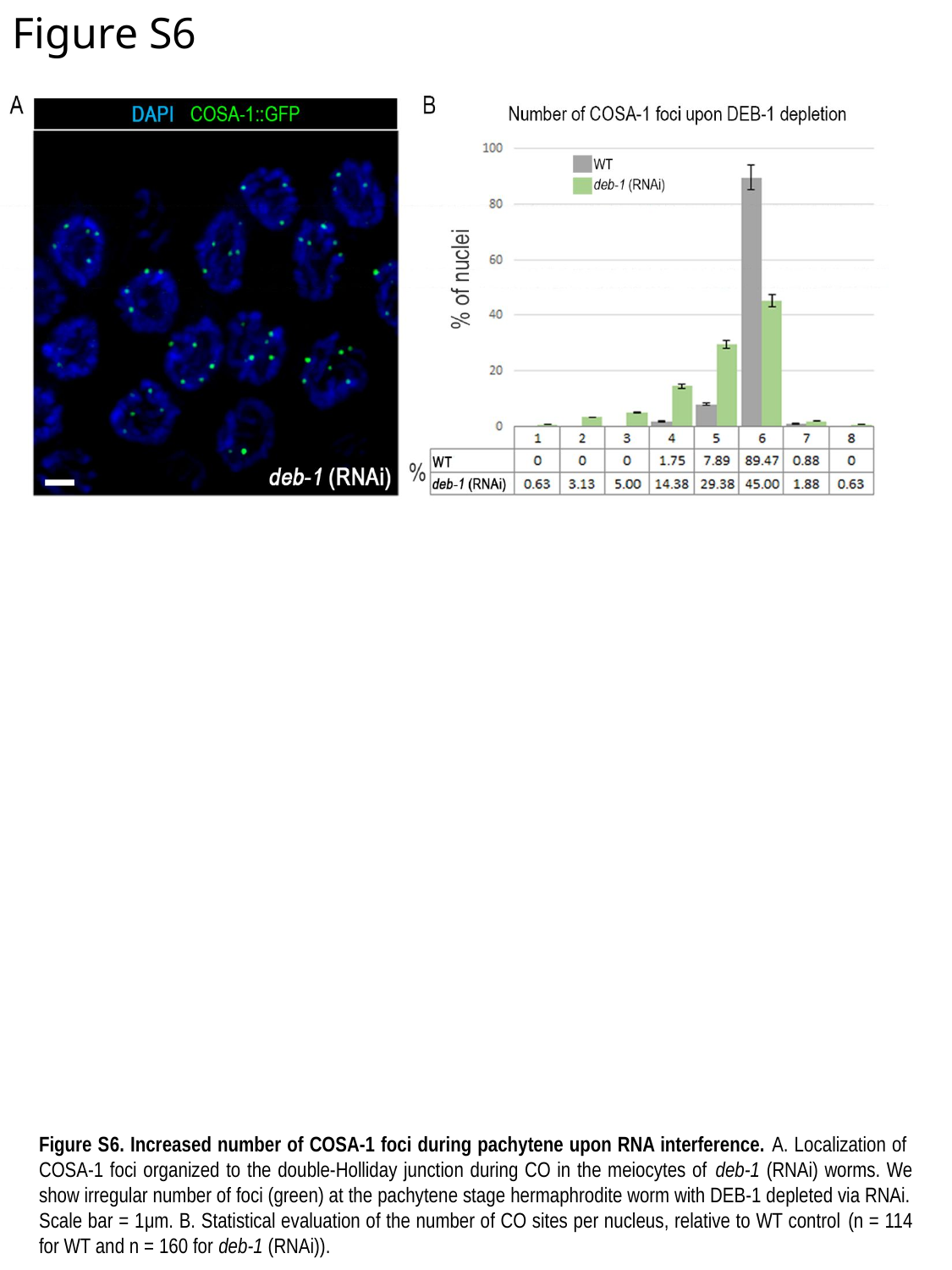

Figure S6
Figure S6. Increased number of COSA-1 foci during pachytene upon RNA interference. A. Localization of COSA-1 foci organized to the double-Holliday junction during CO in the meiocytes of deb-1 (RNAi) worms. We show irregular number of foci (green) at the pachytene stage hermaphrodite worm with DEB-1 depleted via RNAi. Scale bar = 1μm. B. Statistical evaluation of the number of CO sites per nucleus, relative to WT control (n = 114 for WT and n = 160 for deb-1 (RNAi)).

## Slide 7
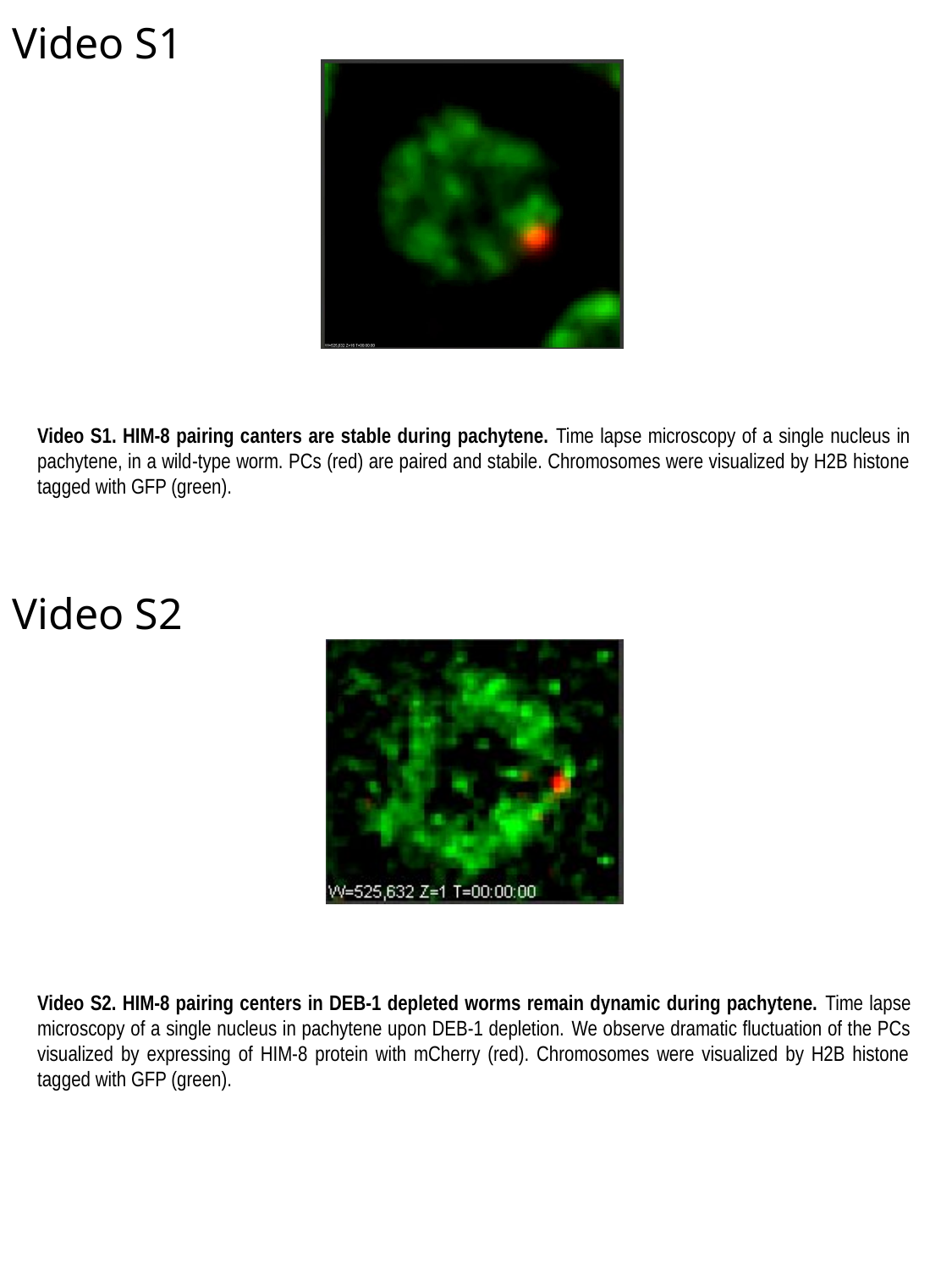

Video S1
Video S1. HIM-8 pairing canters are stable during pachytene. Time lapse microscopy of a single nucleus in pachytene, in a wild-type worm. PCs (red) are paired and stabile. Chromosomes were visualized by H2B histone tagged with GFP (green).
Video S2
Video S2. HIM-8 pairing centers in DEB-1 depleted worms remain dynamic during pachytene. Time lapse microscopy of a single nucleus in pachytene upon DEB-1 depletion. We observe dramatic fluctuation of the PCs visualized by expressing of HIM-8 protein with mCherry (red). Chromosomes were visualized by H2B histone tagged with GFP (green).
